# Hyperelastic Regularization for Near-Diffeomorphic Transformer-Based Brain MRI Registration

**DOI:** 10.3390/jimaging12070276

**Published:** 2026-06-24

**Authors:** Shiyi Xu, Mohan Xu, Erjin Zhou

**Affiliations:** 1 Capital Medical University Second Clinical School, Capital Medical University, Beijing 100050, China; xsy4831@mail.ccmu.edu.cn; 2 School of Physics, Peking University, Beijing 100871, China; 2200011601@stu.pku.edu.cn; 3 Dexmal, Beijing 100096, China

**Keywords:** deformable image registration, brain MRI, TransMorph, Jacobian determinant, Jacobian-aware regularization, hyperelastic regularization, folding suppression, diffeomorphic registration

## Abstract

Transformer-based deformable brain MRI registration achieves high overlap accuracy, but predicted displacement fields can contain voxels with a non-positive Jacobian determinant—local foldings that violate the diffeomorphism assumption required by tensor-based morphometry and atlas-fusion segmentation workflows. We introduce HypEReg, a non-linear hyperelastic regularizer that acts directly on the Jacobian determinant of the predicted displacement field. HypEReg couples a clamped-rational volume-distortion penalty ${\left(\det J_{\phi }-1\right)}^{2}/\max\left(\det J_{\phi },\epsilon \right)$ with an explicit per-voxel anti-folding hinge ${\left[\max\left(0,\epsilon -\det J_{\phi }\right)\right]}^{2}$, integrated as a purely loss-side module into a TransMorph backbone with no inference-graph modifications. On the IXI atlas-to-subject benchmark (115 test subjects), HypEReg-TransMorph maintains grouped Dice (0.7537) while reducing the $\det(J_{\phi })\leq 0$ voxel ratio from $1.502\times 10^{-2}$ (TransMorph) to $1.5\times 10^{-5}$, with identical per-case runtime and parameter count to the unregularized baseline. In strict zero-shot transfer to OASIS Learn2Reg test pairs (no fine-tuning), HypEReg-TransMorph achieves Dice 0.7756 with a $\det(J_{\phi })\leq 0$ ratio of $7.6\times 10^{-5}$, roughly two orders of magnitude below plain TransMorph zero-shot (Dice 0.7691; ratio $9.6\times 10^{-3}$); downstream multi-atlas label fusion further confirms the practical benefit of fold suppression (fused Dice 0.8271 vs. 0.8201 for TransMorph). OASIS-2 longitudinal and ROI analyses support deformation plausibility (lower folding/SDlogJ and stronger ventricular ROI agreement), while clinical-covariate associations remain exploratory rather than biomarker-validating. Determinant-level, non-linear hyperelastic regularization substantially suppresses folding in Transformer dense-flow brain MRI registration while preserving alignment accuracy and adding zero inference cost, providing a practical drop-in regularization strategy that improves the reliability of deformation fields for morphometry-oriented deformable registration.

## 1. Introduction

Deformable registration is a foundational primitive of computational neuroimaging. Atlas propagation, voxel-wise morphometry, and longitudinal monitoring of disease progression all rely on the assumption that a smooth, invertible mapping can be recovered between moving anatomy and a target reference. Classical iterative formulations, exemplified by SyN and other diffeomorphic frameworks, build this assumption into the optimization objective by explicitly constraining transformation smoothness and invertibility [1,2,3]. The deformation fields that these methods produce are well behaved, but each registration is solved as an iterative inverse problem and is therefore expensive to deploy at population scale.

Learning-based registration was introduced largely to remove this throughput bottleneck. Following the introduction of VoxelMorph and related convolutional architectures [4,5], a single forward pass through a trained network can produce a dense displacement field in a fraction of a second, with overlap accuracy that often matches or exceeds classical baselines. Probabilistic diffeomorphic variants further stabilize the predicted deformations [6], and the recent adoption of Transformer encoders has improved long-range contextual modeling for volumetric registration [7,8]. Across these advances, however, one issue has proved persistent: when displacement is predicted directly and without an intrinsic regularity prior, the resulting deformation is not guaranteed to be diffeomorphic. Local foldings—voxels at which $\det J_{\phi }\leq 0$—appear systematically in the output of unconstrained Transformer registration networks, regardless of how high the Dice score happens to be.

This is more than a mathematical inconvenience. The Jacobian determinant is itself the quantity reported in tensor-based morphometry (TBM) and longitudinal volume-change studies [3,9] and is central to imaging endpoints in multiple neuroimaging workflows. When local foldings ($\det J_{\phi }\leq 0$) appear in a deformation field, corresponding voxels report negative or undefined volume change, biasing voxel-wise statistical maps. Constraining the predicted deformation to be near-diffeomorphic at training time therefore has direct implications for the trustworthiness of Jacobian-based analyses.

The deep-learning literature has approached this regularity problem from several angles. Mok and Chung [10] introduced a selective Jacobian-determinant penalty that activates only on candidate folded voxels (detJ≤0) inside a diffeomorphic CNN; this is the conceptual antecedent of the anti-folding term used in the present work. Hering et al. [11] embedded a Burger–Modersitzki–Ruthotto-style volume-change penalty alongside a curvature regularizer in a CNN-based lung-CT registration pipeline, and Zou et al. [12] explored conformal-invariant hyperelastic energies in the same lung-CT setting. Inverse-consistency formulations such as ICON [13] and GradICON [14] encourage cycle agreement between forward and backward warps, while hierarchical constructions such as LapIRN [15] compose the deformation across multiple Laplacian scales. Classical diffeomorphic registration integrates stationary velocity fields by scaling-and-squaring [6,16]; recent Transformer variants extend this idea through velocity-field integration (TransMorph-Diff, TransMorph-TVF) [7,17] or spatially varying regularization (TM-SPR) [18]. These methods impose invertibility structurally or through integration, whereas HypEReg is a soft, empirical topology-control penalty on a direct displacement field. To our knowledge, coupling a non-linear determinant-level hyperelastic-style regularizer of the HypEReg form with a Transformer-based dense-flow backbone and subject-level paired inferential statistics, as we do here, has not been previously reported; it is also in this high-capacity setting that folding artefacts are often most visible.

In this paper, we develop HypEReg, a non-linear hyperelastic regularizer derived from the Burger–Modersitzki–Ruthotto (BMR) energy [19] but redesigned for stochastic optimization in a deep-learning context. HypEReg combines a clamped-rational volume-distortion penalty ${\left(\det J_{\phi }-1\right)}^{2}/\max\left(\det J_{\phi },\epsilon \right)$ with an explicit per-voxel anti-folding hinge ${\left[\max\left(0,\epsilon -\det J_{\phi }\right)\right]}^{2}$, both expressed directly as functions of the Jacobian determinant of the predicted displacement field. The clamped-rational form is bounded under stochastic gradient descent and non-linear in $\det J_{\phi }$ so that compressive deformations tend to be penalized more strongly than expansive ones while the ϵ-clamped formulation helps keep gradients finite across a wide range of local strains; the hinge applies a per-voxel quadratic penalty that grows as $\det J_{\phi }$ approaches the folding threshold. This construction emphasizes explicit, determinant-level control rather than the conformal-invariant energy of Zou et al. [12] or the BMR volume-plus-curvature regularizer of Hering et al. [11], and it extends the selective Jacobian penalty of Mok and Chung [10]—which contributes only the hinge component—with a non-linear volume-distortion term. Because HypEReg is integrated into a TransMorph backbone as a purely loss-side module, the inference graph, runtime, and parameter count are identical to the unregularized baseline, making it a drop-in regularization strategy for Transformer-based dense-displacement registration. We evaluate the resulting HypEReg-TransMorph on 115 held-out IXI atlas-to-subject pairs against nine competing methods with subject-level paired inference (Wilcoxon signed-rank, Benjamini–Hochberg FDR) and further assess cross-cohort generalization via a strict zero-shot transfer to OASIS Learn2Reg test pairs, with downstream multi-atlas label fusion as an application-level proxy for fold-free deformation quality.

The remainder of the paper is organized as follows. Section 2 describes the data, the backbone, the proposed regularizer, and the evaluation protocol. Section 3 reports the IXI benchmark, the HypEReg term-design rationale, the IXI→OASIS zero-shot evaluation, downstream multi-atlas fusion, and runtime profiling. Section 4 discusses the position of HypEReg within the deep-registration regularization literature, its clinical relevance, and the principal limitations of the present study, and Section 5 concludes.

## 2. Materials and Methods

### 2.1. Dataset and Preprocessing

Experiments used the IXI brain MRI dataset [20] under an atlas-to-subject registration protocol. The 576 preprocessed subjects (plus the atlas) were partitioned into 403 training, 58 validation, and 115 test cases; all reported metrics were computed on the 115 held-out test subjects. We used the T1-weighted preprocessed IXI package commonly adopted by TransMorph-style studies [5,7], in which preprocessing comprises skull stripping, affine alignment, and subcortical segmentation generation; the volumes are represented in a common template space and cropped/resampled to 160×192×224. A single fixed atlas image–label pair was used across all compared methods. Intensity images were warped with trilinear interpolation and label maps with nearest-neighbor interpolation. The protocol is summarized in Table 1.

#### 2.1.1. OASIS Cross-Cohort Protocols (Zero-Shot and In-Cohort Retraining)

To probe cohort shift beyond IXI, we evaluated structural brain MRI from the Open Access Series of Imaging Studies (OASIS) [21], following the Learn2Reg cross-subject registration protocol for OASIS [22]. Primary volumes were obtained under the terms of the public OASIS archive (https://www.oasis-brains.org/ (accessed on 11 April 2026)); preprocessing and pair definitions follow that benchmark definition [22]. We distinguish two OASIS protocols. (i) Strict IXI→OASIS zero-shot transfer: IXI-trained checkpoints (including HypEReg-TransMorph) were evaluated on the released OASIS test pairs without any fine-tuning on OASIS; displacement fields are exported at half resolution and scored with the same Jacobian and surface-metric pipeline as the IXI benchmark. The main-text OASIS results report grouped Dice, HD95, ASSD, non-positive Jacobian ratio, and SDlogJ (Section 3.4 and downstream fusion in Section 3.7); additional intensity-image scores are not tabulated here. Classical SyN (ANTs) and affine mutual-information baselines were run through the same adapter style as on IXI. Downstream multi-atlas fusion uses these same IXI-trained checkpoints. (ii) In-cohort OASIS retraining (supplementary inferential export): we additionally trained HypEReg-TransMorph on the released OASIS train/validation splits using the same TransMorph backbone and HypEReg operating point $\left(\beta ,\gamma ,\epsilon \right)=\left(0.02,20,10^{-3}\right)$ as on IXI. The resulting OASIS-retrained checkpoint was evaluated on the same n=19 test pairs; Supplementary Table S6 summarizes paired Wilcoxon + Benjamini–Hochberg FDR contrasts against selected baselines for that protocol. The means in Supplementary Table S6 (e.g., grouped-mean Dice ≈0.797 for the OASIS-retrained HypEReg model in our export) are not interchangeable with the strict zero-shot means reported in Section 3.4 (e.g., grouped-mean Dice 0.7756 for IXI-trained HypEReg-TransMorph).

#### 2.1.2. OASIS-2 Longitudinal Morphometry Protocol

To test whether Jacobian regularity supports trustworthy morphometric readouts beyond cross-subject overlap, we additionally analyzed the OASIS longitudinal release (150 subjects, 373 sessions; native CDR, MMSE, and normalized whole-brain volume, nWBV) [23]. Raw T1 volumes were skull-stripped, bias-corrected, and resampled to 160×192×224; for each subject we formed consecutive-visit pairs (n=223 intervals) and registered the later visit to the earlier visit using *IXI-trained* HypEReg-TransMorph and plain TransMorph checkpoints (three seeds each) under strict zero-shot transfer. To reduce pose/global confounding in longitudinal Jacobian readouts, moving visits were rigidly pre-aligned to fixed visits before deformable inference; downstream Jacobian metrics were computed on the resulting relative deformation fields. Brain-mask mean $\log\det J_{\phi }$ is compared to ΔnWBV per year at subject level (Pearson/Spearman, calibrated MAE/RMSE, sign agreement). Clinical associations use subject-level mean atrophy rate versus age, MMSE, and CDR (linear slopes), and between-model differences are tested with a CDR×model interaction term. Fine anatomical ROI plausibility uses FastSurfer-derived OASIS-2 segmentations [24,25], which reproduce the FreeSurfer automated subcortical labeling protocol [26], under strict IXI→OASIS zero-shot transfer. Section 3.8 reports these endpoints.

### 2.2. Baseline Models

The base architecture is TransMorph, a Transformer-based dense deformable registration network with a Swin-style encoder and a CNN decoder that predicts the displacement field [7,8]. We compare HypEReg-TransMorph against deep-learning and classical baselines under identical preprocessing and evaluation scripts. The Transformer family includes TransMorph [7], the Bayesian variant TransMorphBayes from [7], the diffeomorphic velocity-field-integration variant TransMorph-Diff (scaling-and-squaring of a predicted stationary velocity field on the TransMorph backbone) [7], CoTr [27], nnFormer [28], and PVT [29]; the CNN/hybrid family includes VoxelMorph-1 [4], CycleMorph [5], and MIDIR [30]; the classical reference is SyN (ANTs) [1]. TransMorph-Diff and TransMorph-TVF [17] provide velocity-field-integration references rooted in scaling-and-squaring [16]; MIDIR provides a B-spline fold-free reference [30]. Together they contrast structural invertibility constraints with HypEReg’s loss-side regularity. Checkpoint and version identifiers are documented in the repository evaluation configuration and in the supplementary checkpoint manifest (Supplementary Table S1). For consistency, the baseline checkpoints used in our experiments are taken from the TransMorph paper’s official GitHub release (which aggregates links/files for the compared methods) [31] and were evaluated under the present 115-subject IXI test split using a single shared atlas, preprocessing pipeline, evaluation script, and metric implementation. The 403/58/115 split adopted here matches the split distributed with the public TransMorph preprocessed IXI package and is the convention adopted by the IXI atlas-to-subject literature, so the held-out test set is consistent across compared methods; the upstream training-split provenance of each baseline checkpoint, together with its model-card reference, is recorded in the reproducibility notes.

### 2.3. Proposed HypEReg-TransMorph Framework

HypEReg-TransMorph augments the TransMorph baseline with explicit hyperelastic regularization that constrains physically implausible local distortion. Two complementary aspects of deformation quality are targeted: moderation of local volume change, and active suppression of folding near singular Jacobian regions. The general mathematical formulation also includes a length-change term, but its coefficient is set to zero in the operating point because the standard squared first-order finite-difference smoothness penalty $L_{\mathrm{grad}}$ already controls displacement smoothness on the same spatial scale.

**Figure 1:**
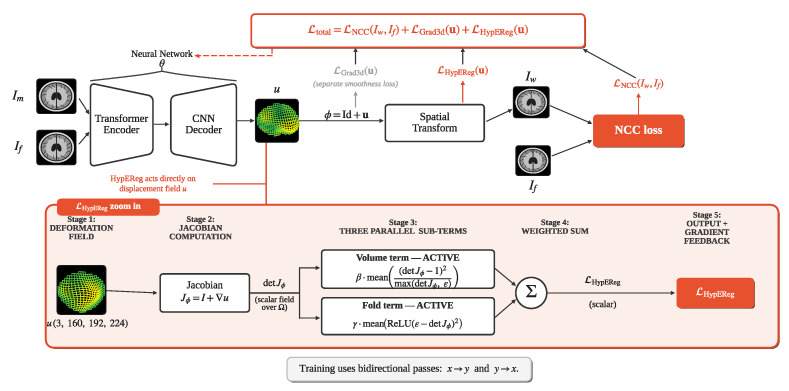
Architecture and training objective of the proposed HypEReg-TransMorph framework. The moving image ($I_{m}$) and fixed image ($I_{f}$) are concatenated and passed through a TransMorph-based registration network with a Transformer encoder and CNN decoder to predict a dense 3D displacement field (*u*). The deformation transform is defined as ϕ=Id+u, and a spatial transformer warps the moving image to generate the warped image ($I_{w}$). During training, three complementary terms are optimized: similarity loss $L_{\mathrm{sim}}\left(I_{w},I_{f}\right)$, smoothness $L_{\mathrm{grad}}\left(u\right)$, and hyperelastic regularization $L_{\mathrm{HypEReg}}\left(u\right)$, with $L_{\mathrm{sim}}=-\bar{{\mathrm{LCC}}^{\;2}}\left(I_{w},I_{f}\right)$. The HypEReg zoom-in panel details computation of $L_{\mathrm{HypEReg}}$: $u\to J_{\phi }=I+\nabla u\to \det J_{\phi }\to$ volume/folding sub-terms. Volume and folding terms are weighted with β=0.02 and γ=20, respectively. Solid arrows denote forward computation and loss aggregation; the dashed orange arrow denotes gradient feedback from L to network parameters (θ). Training uses bidirectional passes (x→y, y→x).

The training architecture is summarized in Figure 1. The moving image $I_{m}$ and fixed image $I_{f}$ are concatenated and passed through the TransMorph-style backbone, which produces a dense displacement field *u*. The deformation is parameterized as ϕ=Id+u, and a spatial transformer [32] warps $I_{m}$ to obtain $I_{w}$. The training loss aggregates similarity $L_{\mathrm{sim}}\left(I_{w},I_{f}\right)$, smoothness $L_{\mathrm{grad}}\left(u\right)$, and hyperelastic regularization $L_{\mathrm{HypEReg}}\left(u\right)$ into a single objective. The training-time HypEReg path evaluates $J_{\phi }=I+\nabla u$ and $\det J_{\phi }$, from which the volume-distortion and anti-folding penalties are formed. We use β=0.02 and γ=20 at the operating point. The network is trained with directional symmetry updates (x→y) and (y→x); each direction performs its own forward/backward/optimizer step.

### 2.4. Loss Function

The deformation is ϕ(x)=x+u(x), with $u:\Omega \to R^{3}$ the dense displacement field. The unified training objective is

(1) $$L_{\mathrm{total}}=L_{\mathrm{sim}}+\lambda_{\mathrm{grad}}L_{\mathrm{grad}}+L_{\mathrm{HypEReg}},$$

where the HypEReg regularization term decomposes into a volume-distortion penalty and an anti-folding hinge:

(2) $$L_{\mathrm{HypEReg}}\left(u\right)=\beta \;L_{\mathrm{volume}}\left(u\right)+\gamma \;L_{\mathrm{fold}}\left(u\right).$$

The similarity loss $L_{\mathrm{sim}}=-\bar{{\mathrm{LCC}}^{\;2}}\left(I_{w},I_{f}\right)$ is the negative mean squared local correlation between the warped and fixed images, evaluated with a $9^{3}$ sliding window as in the VoxelMorph reference implementation [4] (the form commonly referred to as “local NCC” in the deep registration literature). The smoothness term is the squared first-order finite-difference penalty

(3) $$L_{\mathrm{grad}}\left(u\right)=\frac{1}{3}\left(\bar{{|\partial_{x}u|}^{\;2}}+\bar{{|\partial_{y}u|}^{\;2}}+\bar{{|\partial_{z}u|}^{\;2}}\right),$$

where the overline denotes voxel-wise and channel-wise mean and $\partial_{d}$ is the forward finite difference along axis *d*. The Jacobian $J_{\phi }\left(x\right)=I+\nabla u\left(x\right)$ is computed with forward finite differences on interior voxels (D−1,H−1,W−1 stencil). For the 160×192×224 field size, the interior mask covers 98.416% of voxels and excludes 1.584% of the boundary voxels; the same Jacobian implementation and interior mask are used for all learned models. The two HypEReg sub-terms in Equation (2) are

(4) $$L_{\mathrm{volume}}\left(\varphi \right)=\frac{1}{\left|\Omega \right|}\sum_{x\in \Omega }\frac{{\left(\det J_{\varphi }\left(x\right)-1\right)}^{2}}{\max(\det J_{\varphi }\left(x\right),\epsilon )},$$

(5) $$L_{\mathrm{fold}}\left(\varphi \right)=\frac{1}{\left|\Omega \right|}\sum_{x\in \Omega }{\left[\max(0,\epsilon -\det J_{\varphi }\left(x\right))\right]}^{2}.$$

#### 2.4.1. Relation to the Burger–Modersitzki–Ruthotto (BMR) Hyperelastic Energy

HypEReg is a deep-learning instantiation of the BMR hyperelastic family [19], redesigned for stochastic optimization and for direct compatibility with intensity-only similarity losses. Four design choices distinguish it from the original variational energy. First, the *volume term* is a clamped-rational form rather than a quartic. BMR use the symmetric quartic $\phi_{v}^{\mathrm{BMR}}\left(v\right)={\left(v-1\right)}^{4}/v^{2}$, which satisfies $\phi_{v}^{\mathrm{BMR}}\left(1/v\right)=\phi_{v}^{\mathrm{BMR}}\left(v\right)$ and diverges as $v\to 0^{+}$. Replacing it with $\phi_{v}^{\mathrm{ours}}\left(v\right)={\left(v-1\right)}^{2}/\max\left(v,\epsilon \right)$, $\epsilon =10^{-3}$, accomplishes three goals simultaneously: the penalty is bounded above by 1/ϵ and therefore yields finite, well-conditioned gradients under stochastic gradient descent; it remains strictly asymmetric in v↔1/v, so compressive ($\det J_{\phi }\to 0^{+}$) deformations are still penalized more strongly than expansive ones, preserving the BMR “shrinkage is more expensive” inductive bias; and it is non-linear in $\det J_{\phi }$ and therefore remains well-conditioned at moderate-to-large local strains under ϵ-clamping, without requiring a small-strain approximation. Second, because $\phi_{v}^{\mathrm{ours}}$ is bounded under ϵ-clamping, we re-introduce the BMR fold-prevention behavior through an explicit hinge $L_{\mathrm{fold}}=\frac{1}{\left|\Omega \right|}\sum_{x}{\left[\max\left(0,\epsilon -\det J_{\phi }\right)\right]}^{2}$. This hinge activates only on candidate folded voxels ($\det J_{\phi }\leq \epsilon$), provides a per-voxel quadratic supervision signal that is zero everywhere else, and is the deep-learning analogue of the selective Jacobian-determinant penalty introduced by Mok and Chung [10]; combined with $\phi_{v}^{\mathrm{ours}}$ it recovers, in the stochastic-optimization regime, the fold-suppression behavior that BMR achieve via the unbounded quartic. Third, the BMR *cofactor (surface) term* is excluded by design. The BMR energy contains an additional surface-area term $\phi_{w}$ defined on cof∇y, required for existence proofs based on Ball’s polyconvexity and for distance measures that depend on ∇y (e.g., the mass-preserving PET setting in Burger et al. [19]). Since our similarity loss is local NCC, which depends only on intensity values and not on ∇y, the surface term carries no informative gradient under the similarity used here and is therefore omitted from the objective. Fourth, the BMR *length (curvature surrogate) term*—a first-order penalty on ${∥\nabla u∥}_{F}^{2}$—is also excluded. In the variational setting, that term controls first-order displacement smoothness, but in our formulation, this role is already filled by the standard squared-$L^{2}$ gradient penalty $L_{\mathrm{grad}}$, which is the smoothness control used in the VoxelMorph and TransMorph reference implementations and acts on exactly the same spatial scale. Including a BMR length term alongside $L_{\mathrm{grad}}$ would supply linearly correlated supervision on identical quantities with no expressive gain; the two are therefore merged into the single $L_{\mathrm{grad}}$ term. HypEReg thus retains only the two BMR components for which the deep-registration objective lacks an existing analogue: the volume-distortion penalty (with the BMR quartic replaced by a clamped-rational form) and the explicit anti-folding hinge.

The resulting regularizer (clamped-rational volume + anti-folding hinge) is therefore a deep-learning-friendly hyperelastic subset of the BMR energy: it preserves the asymmetric volume-control behavior, replaces the unbounded fold-prevention asymptote with an explicit hinge that is differentiable everywhere, and avoids the variational existence-theoretic machinery that is unnecessary in the stochastic-optimization regime.

#### 2.4.2. Hyperparameter Choices

The final hyperparameters are

$$\lambda_{\mathrm{grad}}=1.0,\;\beta =0.02,\;\gamma =20,\;\epsilon =10^{-3}.$$

The smoothness weight $\lambda_{\mathrm{grad}}$ follows the value used in the original TransMorph reference experiments on the IXI atlas-to-subject benchmark [7]. The outer multiplier on the HypEReg block is fixed to one in our implementation; effective regularization strength is therefore governed by (β,γ). When $\det J_{\phi }\leq 0$, the denominator in $L_{\mathrm{volume}}$ is clamped by ϵ, while $L_{\mathrm{fold}}$ remains active through $\max(0,\epsilon -\det J_{\phi })$, so folded regions are penalized by both terms simultaneously. With $\epsilon =10^{-3}$, a folded voxel with $\det J_{\phi }=0$ already contributes $10^{3}$ from the volume term alone, while $L_{\mathrm{fold}}$ adds a hinge penalty that further emphasizes negative-detJ regions. The coefficients (β,γ)=(0.02,20) were selected on the held-out IXI validation subset (58 subjects) using a coarse two-axis grid sweep, choosing the configuration that maximized validation grouped-Dice subject to a non-positive Jacobian ratio below $10^{-4}$; the corresponding qualitative sensitivity behavior is summarized in Section 3.3. The clamping constant $\epsilon =10^{-3}$ appears identically in $L_{\mathrm{volume}}$ (denominator floor) and in $L_{\mathrm{fold}}$ (hinge threshold); a validation sweep over $\epsilon \in \{10^{-2},10^{-3},10^{-4}\}$ shows that $\epsilon =10^{-2}$ and $\epsilon =10^{-3}$ give near-identical validation behavior, whereas $\epsilon =10^{-4}$ degrades sharply, so $\epsilon =10^{-3}$ sits on a stable plateau whose lower boundary lies near $10^{-4}$ (Section 3.3).

### 2.5. Training Details

Each training mini-batch follows Algorithm 1. HypEReg-TransMorph is trained with the Adam optimizer with AMSGrad [33,34], learning rate $1\times 10^{-4}$, weight decay 0, batch size 2, a polynomial-decay learning-rate schedule with exponent 0.9, and 500 epochs of bidirectional updates (x→y and y→x, each with an independent forward/backward/optimizer step). The checkpoint-selection rule is best validation grouped-Dice on the held-out 58-subject IXI validation set; the released checkpoint corresponds exactly to that selection. Following the convention of the upstream TransMorph reference implementation [7], training is stochastic and does not pin a single global random seed; reproducibility of the reported numerical values is therefore tied to the selected checkpoint and deterministic evaluation protocol rather than to bit-identical re-training. To make the resulting subject-level statistics robust to single-run sampling, inferential analysis is performed with paired Wilcoxon signed-rank tests over the 115 held-out test subjects (Section 2.8); these tests are non-parametric and therefore not affected by Gaussianity assumptions. A full-budget multi-seed extension (≥3 re-trainings) is identified in the limitations as a complementary robustness check.
**Algorithm 1** HypEReg-TransMorph training step (per mini-batch)1:Predict displacement *u* from moving/fixed image pair.2:Warp moving image using ϕ(x)=x+u(x) to obtain $I_{w}$.3:Compute $L_{\mathrm{sim}}$, $L_{\mathrm{grad}}$, and HypEReg components from $J_{\phi }$.4:Direction 1 update: evaluate (x→y), compute $L_{\mathrm{sim}}+L_{\mathrm{grad}}+L_{\mathrm{HypEReg}}$, backpropagate, and apply optimizer step.5:Direction 2 update: swap inputs (y→x), recompute the same loss components, backpropagate, and apply optimizer step.6:Record the mean of both directional losses for logging.

The reported runs use a single NVIDIA RTX PRO 6000 Blackwell Workstation Edition (97,887 MiB VRAM, CUDA 12.8); because Blackwell-class GPUs require a recent CUDA path, training and profiling were executed under PyTorch 2.12 [35]. Reproduction requires Python 3.12.10, PyTorch 2.12.0.dev20260402+cu128, NumPy 2.3.5, antspyx 0.6.3, and SimpleITK 2.5.4. PyTorch 2.12 is therefore a hardware-availability artefact rather than a method dependency. Baselines are evaluated under the same test protocol; classical baselines use ANTs/ANTsPyX and SimpleITK implementations [1,36,37].

### 2.6. Evaluation Metrics

The primary numerical contrasts in Table 2 use grouped mean Dice over 17 VOI groups (higher is better), HD95 and ASSD in millimetres (lower is better), the non-positive Jacobian determinant ratio $\#\{x:\det J_{\phi }\left(x\right)\leq 0\}/\#\left\{x\right\}$ (lower is better), and SDlogJ, ${\mathrm{std}}_{x}\left(\log\max\left(\det J_{\phi }\left(x\right),\epsilon \right)\right)$ (lower is better) [22]. Supplementary Table S2 reports auxiliary descriptors for a focused model subset that use the same evaluation code path but are omitted from Table 2 for space: NSD@1 mm [38], voxel-averaged bending energy, mean absolute divergence of the displacement field [15,39], and Jacobian tail quantiles $\left(J_{\min},J_{p01},J_{p99},J_{\max}\right)$. Training uses local squared cross-correlation as $L_{\mathrm{sim}}$ (Section 2.4).

**Figure 2:**
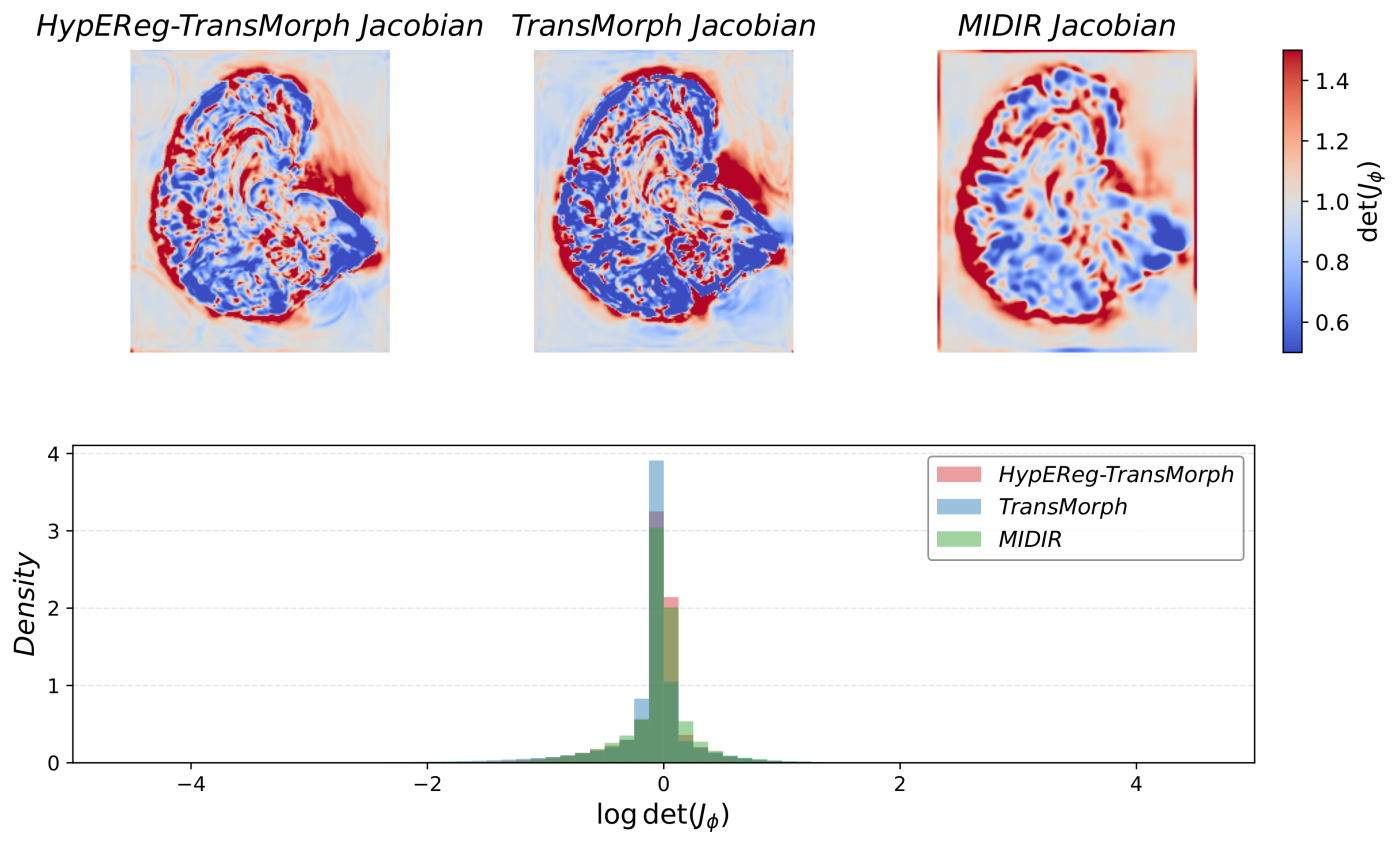
Jacobian-determinant analysis for deformation plausibility. Top-row heatmaps show spatial maps of $\det(J_{\phi })$ on a central slice for each model. The color bar is fixed to the range [0.5,1.5]* across all panels* so that all methods are rendered on a common, comparable scale; values near 1 indicate near-volume-preserving local transforms, values below 0.5 indicate strong local compression, and values above 1.5 indicate strong local expansion (saturated for visualization). The chosen range covers ≥99% of voxels for every method shown and is therefore representative; the full per-method log-Jacobian distribution is reported as a histogram on the bottom row, where narrower distributions centered near zero indicate more stable local volume behavior. The unsaturated extreme-tail statistics ($J_{\min},J_{p01},J_{p99},J_{\max}$) are reported numerically in the supplementary metric tables and form the basis of the SDlogJ values in Table 2, so the visualization range is presentational rather than load-bearing for any quantitative claim.

Figure 2 summarizes Jacobian-determinant trends for deformation plausibility; Supplementary Figure S1 summarizes the broader multi-metric overview.

### 2.7. Grouped-VOI Label Protocol

Grouped Dice uses a 46-label FreeSurfer-compatible index set [26], with labels aggregated into 17 bilateral or anatomically related groups. The explicit group-to-label-ID mapping is listed in Supplementary Table S5.

### 2.8. Statistical Analysis

Inferential analysis is performed with paired Wilcoxon signed-rank tests [40] and Benjamini–Hochberg FDR correction [41] on subject-level outputs from models with complete paired evaluation data. Supplementary Table S3 summarizes mean ± standard deviation contrasts for selected baselines alongside aligned median tests; Supplementary Table S4 reports paired median differences, raw *p*-values, FDR-adjusted *q*-values, and signed effects. Signed effect is defined as the matched-pairs rank-biserial correlation, $r_{\mathrm{rb}}=\left(W_{+}-W_{-}\right)/\left(W_{+}+W_{-}\right)$, computed on metric-aligned paired differences. We additionally report 95% bootstrap confidence intervals on subject-level metric means using B=10,000 resamples (with replacement) of the 115 held-out IXI test subjects with fixed random seed 0. Supplementary Table S7 reports these intervals for grouped Dice, non-positive Jacobian ratio, SDlogJ, HD95, and ASSD for five core models (HypEReg-TransMorph, TransMorph, TransMorphBayes, MIDIR, and SyN), providing uncertainty bounds that are consistent with the Wilcoxon-based paired contrasts.

## 3. Results

### 3.1. Overlap Accuracy on the IXI Benchmark

HypEReg-TransMorph remains competitive on grouped-structure overlap while improving deformation regularity relative to unconstrained Transformer baselines. Compared with the strongest TransMorph-family baseline (TransMorphBayes), grouped Dice changes from 0.7530 to 0.7537, HD95 from 5.7246 mm to 5.3234 mm, and ASSD from 1.4160 mm to 1.3570 mm (all from per-case evaluation exports; see Table 2 and Bootstrap CI in Supplementary Table S7).

### 3.2. Deformation Regularity and Folding Suppression

The principal differences between HypEReg-TransMorph and the unregularized Transformer baselines appear in deformation-regularity metrics. HypEReg-TransMorph yields a non-positive Jacobian determinant ratio of 0.000015±0.000007, compared with 0.015634±0.003363 for TransMorphBayes and 0.015021±0.003416 for TransMorph. SDlogJ decreases from 0.4920±0.0330 (TransMorphBayes) and 0.5064±0.0250 (TransMorph) to 0.3280±0.0221 (HypEReg-TransMorph); for Jacobian upper-tail distortion, $J_{\max}$ drops from 46.0976 (TransMorphBayes; Supplementary Table S2) to 18.6155.

As an explicitly diffeomorphic reference point, we additionally evaluate TransMorph-Diff, a velocity-field-integration variant of the same backbone in which the network predicts a stationary velocity field that is exponentiated by scaling-and-squaring [6,7,16] (Table 2). By construction this baseline is fold-free (non-positive Jacobian ratio =0) and attains the lowest SDlogJ (0.0048±0.0003) in the benchmark. However, this structural regularity comes at a large accuracy cost: grouped Dice falls to 0.5943±0.0455 and surface error rises sharply (HD95 8.6391±1.0670 mm, ASSD 2.4632±0.3380 mm), the worst overlap and surface metrics among all learned models. This contrast is instructive: enforcing diffeomorphism through velocity-field integration on this Transformer backbone over-smooths the deformation and sacrifices correspondence, whereas HypEReg-TransMorph attains near-diffeomorphic regularity (non-positive Jacobian ratio $1.5\times 10^{-5}$) while preserving the overlap and surface accuracy of the unconstrained backbone. Regularity metrics such as SDlogJ and the folding ratio must therefore be interpreted jointly with accuracy, not in isolation.

To quantify run-to-run (training-seed) variability given the small Dice gap between the Transformer baselines, we retrained both TransMorph and HypEReg-TransMorph from scratch under three global seeds {0,1,2} with identical split, optimizer, schedule, loss weights, and best-validation-Dice checkpoint selection, and scored all six checkpoints with one shared deterministic pipeline (Supplementary Table S12). Training-seed variability is small for both models (Dice across-seed std ≈0.002; TransMorph non-positive-ratio std $\approx 7\times 10^{-5}$), and the two effects sit on very different scales: the Dice difference (HypEReg − TransMorph ≈+0.004 across seeds) is modest and partly within seed-level noise—paired per-seed Wilcoxon tests favor HypEReg for seeds 1 and 2 ($p<10^{-9}$) but not seed 0 (p=0.24)—whereas the regularity improvement is stable and overwhelmingly significant in every seed (non-positive Jacobian ratio reduced ∼830×; SDlogJ reduced ≈0.17; $p\approx 1.3\times 10^{-20}$). This confirms that HypEReg’s contribution is deformation regularity at non-inferior overlap rather than a Dice gain that could be attributed to a single fortunate run.

For the models with complete subject-level per-case exports, the inferential analysis supports these trends. After FDR correction, HypEReg-TransMorph improves regularity over plain TransMorph on both non-positive Jacobian ratio and SDlogJ (both q<0.001). Compared with MIDIR, HypEReg-TransMorph is significantly worse on SDlogJ and non-positive Jacobian ratio (both q<0.001), while the HD95 difference is not significant (q=0.59). Compared with SyN, HypEReg-TransMorph is significantly better on HD95 (q<0.001) but worse on SDlogJ (q<0.001). With the newly uploaded TransMorph and TransMorphBayes checkpoints, an additional paired analysis on Dice and non-positive Jacobian ratio confirms the HypEReg-TransMorph advantages versus both baselines after FDR correction (Supplementary Table S4).

### 3.3. Design Rationale, Ablation Study, and Hyperparameter Sensitivity

The HypEReg objective is a two-term construction, $\beta L_{\mathrm{volume}}+\gamma L_{\mathrm{fold}}$, supplementing the global smoothness penalty $L_{\mathrm{grad}}$. The operating point sets β=0.02, γ=20, and $\epsilon =10^{-3}$. The two terms target mathematically distinct failure modes with nearly disjoint support sets, which motivates their joint inclusion.

The volume-distortion term $L_{\mathrm{volume}}$ is active on the entire interior mask. The bounded rational form ${\left(\det J_{\phi }-1\right)}^{2}/\max\left(\det J_{\phi },\epsilon \right)$ is strictly positive whenever $\det J_{\phi }\neq 1$ and approaches zero only at exact volume preservation. It penalizes both compressive ($\det J_{\phi }\to 0^{+}$) and expansive ($\det J_{\phi }\gg 1$) deviations, with the BMR-style asymmetry preserving stronger shrinkage penalties. Its primary mathematical effect is to tighten the bulk of the log-Jacobian distribution, controlling SDlogJ.

The anti-folding hinge $L_{\mathrm{fold}}={\left[\max\left(0,\epsilon -\det J_{\phi }\right)\right]}^{2}$ has support only on the candidate-folded subset $\left\{x:\det J_{\phi }\left(x\right)\leq \epsilon \right\}$, which occupies at most a small fraction of voxels in unregularized baselines and typically vanishes near convergence. Outside this set the term contributes neither value nor gradient. Its primary effect is therefore elimination of negative-determinant voxels rather than global distribution shaping.

Because the two terms act on largely disjoint regions of $\det J_{\phi }$ space, they provide complementary supervision: $L_{\mathrm{volume}}$ shapes the global distribution, whereas $L_{\mathrm{fold}}$ supplies a localized anti-folding signal. Accordingly, our ablation in Table 3 is *component-wise* and not merely a coefficient sweep: it isolates each loss component by switching it off (volume-term-only with γ=0; fold-hinge-only with β=0) and contrasts these against the combined operating point, in addition to varying the coefficients (β,γ). The third component of the general hyperelastic energy, the length-change term, is held at coefficient zero by design because the first-order smoothness penalty $L_{\mathrm{grad}}$ already controls displacement smoothness on the same spatial scale (Section 2); we therefore report it as a fixed design choice rather than a swept variable. Table 3 quantifies the individual term contributions and coefficient sensitivity on the 58-subject IXI validation split—the same split used to select the operating point, so the entire ablation is free of any test-set feedback—while Supplementary Table S2 reports aligned Jacobian-tail evidence ($J_{\min},J_{p01},J_{p99},J_{\max}$) for representative term-isolation variants.

#### 3.3.1. Role of Each Term

On the validation split (Table 3), the fold-hinge-only configuration (β=0,γ=20) leaves the non-positive Jacobian ratio at $4.49\times 10^{-3}$—roughly two orders of magnitude above the $10^{-4}$ selection threshold and comparable to the unregularized Transformer baselines in Table 2 —with the worst SDlogJ (0.470) and surface metrics (HD95 3.270 mm, ASSD 0.769 mm), confirming that $L_{\mathrm{fold}}$ alone cannot drive near-zero folding. The volume-term-only configuration (β=0.02,γ=0) already suppresses the non-positive Jacobian ratio to $1.97\times 10^{-5}$ and tightens SDlogJ to 0.334: $L_{\mathrm{volume}}$, by penalizing compressive deformations across the entire interior mask, is the primary driver of both global distribution shaping and near-zero folding. Adding the fold hinge at the operating point (β=0.02,γ=20) marginally improves SDlogJ (0.327) at essentially unchanged validation Dice (0.751), consistent with the complementary-supervision rationale.

#### 3.3.2. Coefficient Sensitivity (Full Validation Grid)

Table 3 reports the retained validation grid, including the two failing/under-regularized corners. Across the whole grid, grouped Dice is remarkably flat: it spans only 0.7486–0.7518 (0.32 percentage points, well within one cross-subject standard deviation ≈0.026), so overlap is essentially insensitive to the exact (β,γ) within the regularized region—i.e., a genuinely *broad* optimum rather than a sharp ridge.

Deformation regularity, in contrast, is governed almost entirely by the volume weight β: at fixed γ=20, increasing β through the retained values 0.01,0.02,0.05 lowers the non-positive Jacobian ratio ($4.68\times 10^{-5}\to 1.59\times 10^{-5}\to 0.58\times 10^{-5}$) and SDlogJ (0.352→0.327→0.291). The fold weight γ saturates: at fixed β, changing γ from 20 to 50 yields only marginal, non-monotonic changes in the folding ratio ($4.68\times 10^{-5}\to 5.75\times 10^{-5}$ at β=0.01; $5.83\times 10^{-6}\to 4.64\times 10^{-6}$ at β=0.05) at essentially unchanged Dice. All combined and volume-only configurations drive the non-positive Jacobian ratio one to three orders of magnitude below the $10^{-4}$ selection threshold; only the fold-hinge-only corner fails it.

#### 3.3.3. Operating-Point Selection Rationale

The operating point (β,γ)=(0.02,20) was fixed a priori from an initial coarse two-axis validation sweep—maximizing the validation grouped Dice subject to a non-positive Jacobian ratio below $10^{-4}$—before the expanded grid above was computed and before the test set was unsealed. Within the retained grid, this overlap optimum remains broad and flat: heavier volume weighting improves regularity, but its Dice difference from the operating point in grouped Dice ( 0.7514 vs. 0.7510) is far inside one cross-subject standard deviation, and all regularized cells already clear the folding threshold by one to three orders of magnitude. We therefore deliberately retain the moderate, pre-specified operating point rather than retrospectively switching to a heavier-regularization cell; this keeps the reported configuration free of any grid-search-on-held-out-data effect and avoids unnecessary over-regularization. If anything, the grid indicates that a heavier volume weight would suppress folding still further at equal overlap, so the headline test results reported at (0.02,20) (Table 2) are a conservative rather than a favorable operating choice. The clamping constant ϵ appears identically in both sub-terms; a validation sweep over $\epsilon \in \{10^{-2},10^{-3},10^{-4}\}$ shows that $10^{-2}$ and $10^{-3}$ yield near-identical results while $10^{-4}$ degrades sharply, so the chosen $\epsilon =10^{-3}$ lies on a stable plateau bounded below near $10^{-4}$.

### 3.4. IXI→OASIS Zero-Shot Cross-Cohort Generalization

To quantify how the IXI-trained models transfer to OASIS without any fine-tuning, we evaluate the complete set of available IXI checkpoints directly on the 19 OASIS Learn2Reg test pairs using the same metric pipeline as the primary OASIS evaluation. Each model’s IXI-trained checkpoint is loaded and run on OASIS pkl inputs without modification, and no OASIS data is used during training. This is a strict zero-shot cross-cohort transfer experiment. Results are summarized in Table 4.

**Figure 3:**
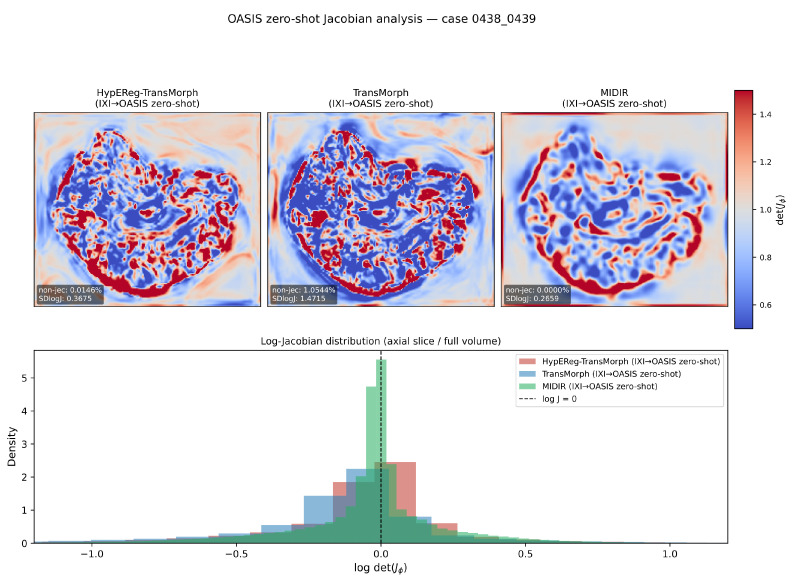
IXI→OASIS zero-shot Jacobian analysis on a representative test pair (fixed = 0438, moving = 0439). **Top row:** spatial maps of $\det(J_{\phi })$ on the central axial slice for HypEReg-TransMorph, TransMorph, and MIDIR; color range fixed to [0.5,1.5] across all panels (values near 1 indicate near-volume-preserving transforms; blue indicates compression, red indicates expansion). Inset text reports per-case non-positive Jacobian ratio and SDlogJ. **Bottom row:** log-Jacobian distribution histogram over the full volume for all three models; the dashed line marks $\log\det J_{\phi }=0$. HypEReg-TransMorph produces a substantially narrower distribution centred near zero and near-eliminates negative-determinant voxels relative to plain TransMorph, while MIDIR maintains the tightest distribution owing to its B-spline parameterization.

Among IXI-trained Transformer models, HypEReg-TransMorph achieves the highest Dice (0.7756) and the lowest non-positive Jacobian ratio ($7.6\times 10^{-5}\pm 3.9\times 10^{-5}$) within the zero-shot group—roughly two orders of magnitude below plain TransMorph zero-shot ($9.6\times 10^{-3}$). The folding-suppression effect of HypEReg therefore generalizes to the unseen OASIS distribution without fine-tuning. TransMorph (zero-shot, 0.7691 Dice) and TransMorphBayes (zero-shot, 0.7587 Dice) transfer competitively above the CNN baselines CycleMorph and VoxelMorph-1, suggesting that Transformer-based dense-flow models carry IXI representations that are broadly useful on OASIS. PVT transfers least well (0.6360 Dice, 1.62% non-positive Jacobian ratio), likely reflecting the larger architecture mismatch between its pyramid-vision-transformer inductive bias and the OASIS contrast/anatomy distribution. MIDIR achieves the best SDlogJ (0.2551) and zero non-positive Jacobian ratio among learned methods, owing to its B-spline parameterization, which structurally precludes folding regardless of the domain. Classical SyN provides a strong deformable reference (0.7385 Dice, near-zero non-positive Jacobian ratio, SDlogJ = 0.2075). Figure 3 visualizes Jacobian distributions on a representative OASIS zero-shot test pair.

The official Learn2Reg OASIS test split provides only n=19 pairs, which limits the precision of the cross-cohort *overlap* estimates above (see Section 4.6). To confirm that the zero-shot *regularity* advantage is not an artefact of this small sample, we additionally evaluated per-ROI Jacobian plausibility on **393 consecutive labeled OASIS cross-subject pairs** (394 subjects) under the same strict zero-shot transfer. In all three atrophy-sensitive ROIs examined (hippocampus, lateral ventricles, cortical ribbon), HypEReg-TransMorph reduces the per-ROI non-positive Jacobian ratio and SDlogJ by roughly one to two orders of magnitude relative to plain TransMorph, with every paired Wilcoxon contrast surviving Benjamini–Hochberg correction at $q<10^{-60}$ (Supplementary Table S14). The folding-suppression benefit therefore holds at ∼20× the official sample size, even though large-*n* overlap labels are not part of the standard benchmark.

### 3.5. Inference Efficiency

Table 5 reports pure forward-pass profiling results for the learned baselines, obtained by timing no-gradient model calls on synthetic inputs of size 1×2×160×192×224 (warm-up + 20 repeated runs; peak CUDA memory). Because HypEReg modifies only the training loss and leaves the inference architecture unchanged, HypEReg-TransMorph achieves *identical* per-case forward runtime (0.0822 s) and peak memory (5.685 GB) to the unregularized TransMorph baseline. Relative to TransMorphBayes (0.0852 s, 6.042 GB, 46.773 M), HypEReg-TransMorph is slightly faster and less memory-intensive, with a nearly identical parameter count. The hyperelastic regularization strategy therefore incurs zero additional inference overhead: the measurable improvement in deformation regularity (non-positive Jacobian ratio reduced by >1000×) is achieved entirely at training time.

Because this benefit is paid for at training time, we also quantify the training-time cost of the added Jacobian computation, which the inference profiling above does not capture. Under matched conditions (single RTX PRO 6000, full input 1×2×160×192×224, cuDNN benchmarking disabled, 40 timed optimization steps after 15 warm-up steps, both models profiled in a single interleaved loop to cancel ordering effects), a full HypEReg-TransMorph training step (forward + backward + optimizer) takes ≈398 ms versus ≈386 ms for the unregularized TransMorph baseline on an otherwise-idle GPU—a per-step overhead of ≈3% (+12 ms). Because the device is shared, absolute step time scales with concurrent load (across repeated runs the baseline step ranged from ≈0.39 to 1.6 s), but the *relative* overhead remained small and stable in the single-digit-percent range (3–7%) across runs. The added peak training memory is negligible (<10 MB; shared peak 13.1 GB), because the HypEReg path only forms the finite-difference Jacobian determinant of the predicted field and two element-wise penalties on the interior voxel mask. Profiled in isolation, the HypEReg loss (Jacobian determinant plus the volume and anti-folding terms, forward and backward) costs ≈13 ms on a 1×3×160×192×224 field, a few percent of a baseline step; the remainder of the per-step overhead is the additional backward flow through the determinant computation. This one-time training overhead does not accumulate at deployment: the trained HypEReg-TransMorph has exactly the inference cost of TransMorph. The profiling script is included in the reproducibility package.

#### Cross-Model Training Cost

Table 6 extends the comparison to the *training* cost of every parametric deep-learning baseline, reporting trainable parameters, forward FLOPs, full training-step (forward + backward) FLOPs, an indicative per-step wall time, and peak training memory for one optimization step on a 1×2×160×192×224 input. Two observations are relevant to HypEReg. First, HypEReg-TransMorph and the unregularized TransMorph have *identical* parameters (46.771 M), forward FLOPs (1447 GFLOPs) and training FLOPs (4323 GFLOPs): the hyperelastic term adds only elementwise Jacobian-determinant and penalty operations on the interior mask, which contribute no convolution/matmul FLOPs and account for the small (∼5%) wall-time difference and <10 MB extra memory reported above rather than any change in the dominant compute. (The indicative step times in Table 6 use a lighter loss harness and therefore differ in absolute terms from the controlled interleaved microbenchmark above, but both yield the same few-percent HypEReg overhead.) Second, this training cost is moderate within the field: it is far below the heaviest Transformer baseline CoTr (∼12.8 TFLOPs/step, 15.9 GB) and comparable to TransMorphBayes, while the lightweight CNN/B-spline models (VoxelMorph-1, CycleMorph, MIDIR) are cheaper to train but do not match HypEReg-TransMorph on the accuracy/regularity trade-off (Table 2). Crucially, none of this training cost is paid at deployment: inference cost is identical to TransMorph (Table 5).

### 3.6. Qualitative Visualization

**Figure 4:**
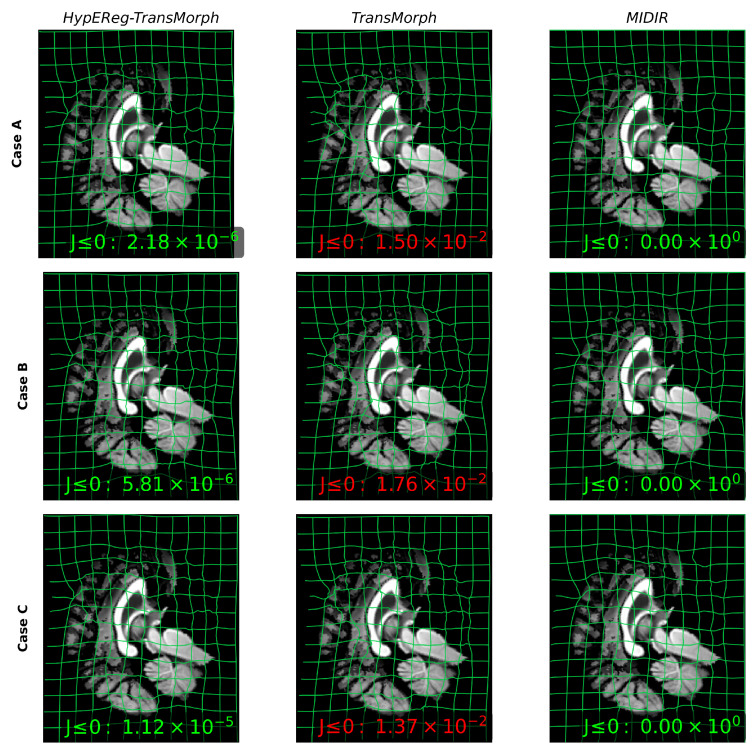
Deformation-grid visualization on three representative IXI test subjects (rows) for HypEReg-TransMorph, TransMorph, and MIDIR (columns). Green lattice lines represent transformed coordinates after warping by each model. Smooth, non-self-intersecting grids indicate stable local geometry; abrupt kinks or crossings indicate folding risk. Each panel shows the central axial slice; the inset label reports the per-case non-positive Jacobian determinant ratio $\#\{x:\det J_{\phi }\left(x\right)\leq 0\}/\#\left\{x\right\}$ (green: ≤$3\times 10^{-4}$; red: ≥$5\times 10^{-3}$). Case A (**top**): subject with the maximum TransMorph/HypEReg folding contrast; Case B (**middle**): high-contrast case at median SDlogJ; Case C (**bottom**): third high-contrast case with lowest HypEReg SDlogJ. MIDIR achieves zero folding via B-spline parameterization; HypEReg-TransMorph reaches near-zero folding through learned Jacobian supervision, with visually comparable grid regularity to MIDIR and notably smoother grid geometry than TransMorph.

Deformation-grid visualization across three representative subjects is shown in Figure 4. Case A is the subject with the highest HypEReg/TransMorph folding contrast (TransMorph non-positive Jacobian ratio $1.50\times 10^{-2}$ vs. HypEReg $2.18\times 10^{-6}$); Case B is the subject from the highest-contrast quartile with the closest-to-median SDlogJ; Case C is a third high-contrast case with the lowest SDlogJ among HypEReg outputs. Across all three cases, HypEReg-TransMorph qualitatively shows smoother and more uniform grid geometry than TransMorph while matching MIDIR in visual regularity. These visual observations are consistent with the quantitative metrics in Table 2. Warped-image registration comparisons across three orthogonal views (axial/coronal/sagittal) are provided as Supplementary Figure S2, complementing the deformation-level evidence presented here. A descriptive multi-metric bar-chart overview (Dice, non-positive Jacobian ratio, SDlogJ, and HD95) is provided as Figure S1 in the Supplementary Materials.

### 3.7. Downstream Multi-Atlas Segmentation on OASIS

Single-atlas Dice (Table 4) measures average correspondence between one warped atlas and a target. Clinical atlas-based segmentation pipelines typically combine *N* atlases by label fusion, in which case the relevant question is how consistently the *N* warped label maps agree at each voxel. Registration models that produce locally inconsistent or folded warps degrade fusion quality even when their per-pair Dice is high.

We evaluate this directly on the OASIS zero-shot setting. Note that Table 4 follows the official 19 predefined Learn2Reg test pairs, whereas multi-atlas fusion is target-centric and therefore uses 20 unique OASIS test targets available for segmentation aggregation in our exported split. Six atlas subjects (IDs 50, 80, 150, 220, 300, 380) are held fixed across all models. For each of the 20 test targets, each atlas is registered to the target with each model; the 35-class OASIS atlas labels are propagated through the predicted displacement field using nearest-neighbor interpolation; and the six warped label maps are fused by majority voting [42]. Per-structure Dice between the fused label map and the target’s ground-truth segmentation is reported, together with the fusion improvement $\Delta \mathrm{Dice}={\mathrm{Dice}}_{\mathrm{fusion}}-{\bar{\mathrm{Dice}}}_{\mathrm{single}}$, which isolates the gain attributable to label fusion from the baseline atlas–target overlap.

**Figure 5:**
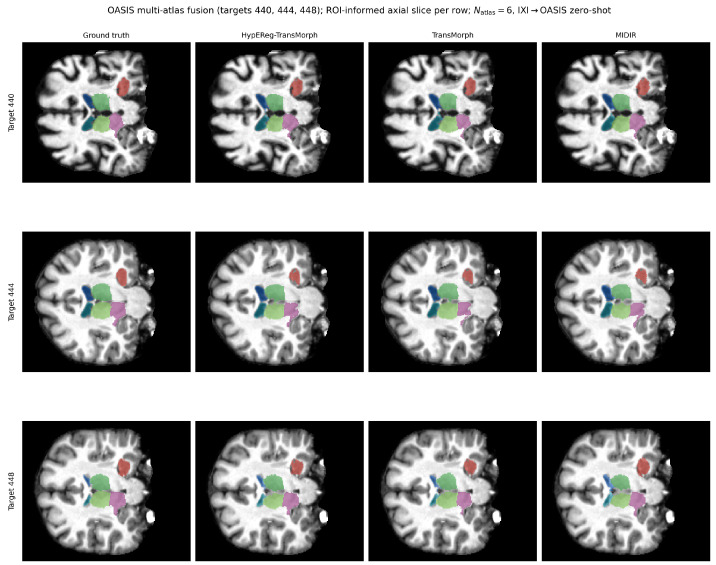
Qualitative downstream comparison on three OASIS test targets (rows) under strict IXI→OASIS zero-shot inference. Columns: ground-truth structures (hippocampus, lateral ventricles, thalamus in distinct colors) and multi-atlas majority-voting fused segmentations ($N_{\mathrm{atlas}}=6$) from HypEReg-TransMorph, plain TransMorph, and MIDIR on the same T1 slice per row (slice chosen to maximize visibility of these ROIs). Fold-regularized HypEReg warps yield fused label maps closer to the reference than the unconstrained Transformer baseline, while MIDIR illustrates a structurally fold-free B-spline parameterization at lower absolute fused overlap (Table 7).

Results are summarized in Table 7 and visualized in Figure 5. HypEReg-TransMorph achieves the highest fused Dice (0.8271±0.0181) in the zero-shot group. Plain TransMorph is second (0.8201±0.0145). MIDIR attains the largest fusion gain (ΔDice=+0.0534) but a lower absolute fused Dice (0.7696±0.0162), consistent with its lower single-atlas baseline (0.7161±0.0141). TransMorphBayes attains 0.8058±0.0206 fused Dice with ΔDice=+0.0460. Per-ROI breakdown shows HypEReg-TransMorph leading on hippocampus (0.8654) and thalamus (0.9227) fused Dice, the two structures most sensitive to local folding artefacts.

### 3.8. Jacobian Morphometry Validation (OASIS-2 Longitudinal and ROI)

The IXI and OASIS cross-subject experiments above establish overlap accuracy, surface accuracy, and local deformation regularity under held-out and zero-shot conditions. These metrics are necessary for evaluating a registration model, but they do not fully test the setting in which the Jacobian determinant is typically used as a morphometric signal: repeated scans of the same subject, where a deformation field is interpreted as a map of anatomical change over time. A model can achieve high Dice and low folding on cross-sectional pairs while still producing longitudinal Jacobian readouts that are weakly related to expected volume-change patterns. We therefore add OASIS-2 to examine whether the regularity advantage of HypEReg remains visible when the predicted deformation is used as a longitudinal volume-change proxy rather than only as an alignment field.

OASIS-2 is useful for this purpose because it provides repeated T1-weighted MRI sessions with native longitudinal variables and enough within-subject intervals to evaluate cohort-shift behavior without retraining on the target cohort. The analysis uses IXI-trained zero-shot checkpoints with three training seeds per model family and rigid pre-alignment before deformable inference, then summarizes three result groups: pair-level deformation regularity, whole-brain volume-change consistency and clinical-covariate association, and ROI-level agreement with FastSurfer-derived anatomical trajectories.

#### 3.8.1. OASIS-2 Longitudinal Volume-Change Consistency

Across 223 consecutive within-subject intervals (150 subjects), HypEReg-TransMorph retains a large Jacobian-regularity advantage over plain TransMorph under strict IXI→OASIS-2 zero-shot transfer (across-seed mean non-positive ratio $1.37\times 10^{-3}\pm 8.20\times 10^{-4}$ vs. $2.11\times 10^{-2}\pm 3.45\times 10^{-3}$; SDlogJ 0.401±0.041 vs. 0.601±0.015; Table 8). This is the primary longitudinal result: the fields from which Jacobian morphometry would be computed are substantially less folded and less dispersed under cohort shift. The brain-mask mean $\log\det J_{\phi }$ versus native ΔnWBV/year is retained only as a coarse global sanity check: subject-level linear correlations are weak for both models, and the fuller supplementary panel shows that individual-level nWBV correlations are mostly non-significant across aggregation schemes (Supplementary Tables S8 and S15). Thus the OASIS-2 longitudinal analysis supports deformation plausibility, not accurate whole-brain linear tracking of nWBV.

#### 3.8.2. OASIS-2 Clinical Covariate Associations

For the whole-brain atrophy proxy, CDR association is weak for both models (HypEReg mean $r_{\mathrm{CDR}}=-0.009\pm 0.034$; TransMorph $r_{\mathrm{CDR}}=-0.079\pm 0.011$), with no robust between-model difference (CDR×model interaction p=0.289). Slopes versus age and MMSE are likewise small and non-significant across seeds. ROI-level CDR/MMSE and CDR >0 versus CDR =0 summaries show weak-to-moderate, regionally plausible ventricular and thalamic trends (Supplementary Table S14), but no corrected between-model clinical interaction. Thus, within this OASIS-2 analysis, the strongest reproducible effect remains deformation regularity rather than clinical-covariate separation.

#### 3.8.3. Validation of the FastSurfer ROI Reference Trajectories

Before using FastSurfer-derived ROI volume-change rates as the anatomical reference for model comparison, we first tested whether these ROI trajectories themselves follow recognized atrophy patterns. This fixed-seed random subset audit used 60 of the 223 longitudinal pairs and repeated three segmentation-only checks that are independent of the registration model: expected atrophy directionality, bilateral consistency, and external agreement with native ΔnWBV/year (Supplementary Table S16). In the 60-pair subset, direction consistency on atrophy pairs (ΔnWBV/year <0) was 95.8% for lateral ventricles (expansion), 79.2% for cerebral cortex (shrinkage), 91.7% for white matter (shrinkage), and 95.8% for hippocampus (shrinkage), all significant in one-sided binomial tests ($p<10^{-4}$). Bilateral left–right annualized rates were strongly correlated (ventricles r=0.888, hippocampus r=0.838; both $p<10^{-15}$), and ventricular expansion also showed strong external consistency with ΔnWBV/year (r=−0.638, $p=4.3\times 10^{-8}$). Full-cohort estimates (n=223) showed the same qualitative pattern. These segmentation-only checks support the anatomical plausibility of the FastSurfer-derived ROI rates used below as reference endpoints.

#### 3.8.4. Anatomical Endpoints and Recognized Atrophy Patterns

**Figure 6:**
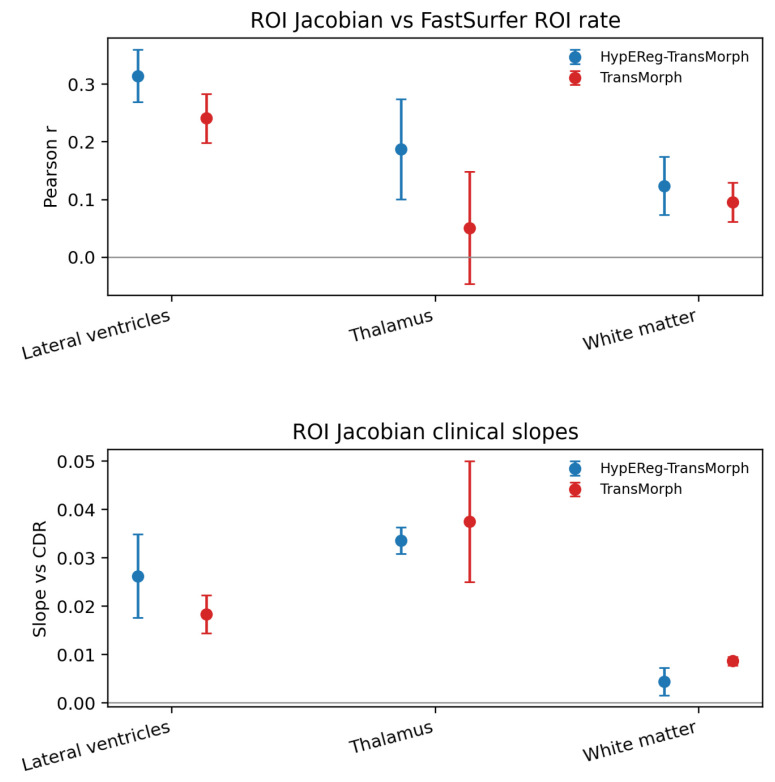
OASIS-2 ROI morphometry validation under IXI→OASIS-2 zero-shot transfer for the three ROI readouts retained in the main-text summary. **Upper:** seed-wise and family-mean Pearson correlations between ROI-integrated Jacobian relative change and FastSurfer ROI volume-change rates. **Lower:** seed-wise and family-mean slopes of ROI Jacobian change versus CDR. Ventricular agreement is the most reproducible endpoint (3/3 significant seeds in both families), while ROI-level CDR between-model interactions are not significant after correction. Numerical values are tabulated in Table 9, with additional OASIS-2 morphometry checks in Supplementary Tables S8, S9 and S14–S16.

Having established the plausibility of the FastSurfer-derived ROI reference trajectories, we then compared model-derived ROI-integrated Jacobian change against those ROI volume-change rates. This provides an anatomical endpoint independent of registration-model identity. The strongest and most reproducible agreement appears in lateral ventricles for both model families, with all 3/3 seeds significant after BH-FDR in both HypEReg and TransMorph. HypEReg shows higher mean ventricular agreement (r=0.315±0.045 vs. 0.241±0.042) and better secondary ROI consistency in thalamus and white matter (Figure 6; Table 9). These results support anatomical consistency of HypEReg-derived Jacobian readouts, although not a confirmed between-model clinical-effect difference.

### 3.9. Topology-Preservation, Inverse Consistency, and Discretization Robustness

HypEReg is a loss-side penalty rather than a structurally constrained parameterization, so it provides no *hard* mathematical guarantee of global invertibility; its assurance is empirical and operates by driving $\det J_{\phi }$ away from the non-positive regime during training. We therefore quantify topology with two checks that go beyond counting $\det J_{\phi }\leq 0$ voxels. First, as an inverse-consistency probe we register A→B and B→A and measure the residual displacement of the composed forward–backward warp (inverse-consistency error, ICE, in voxels) over the 115 IXI test pairs. HypEReg-TransMorph attains the lowest mean ICE (1.66±0.10), well below plain TransMorph (4.21±0.12) and even below the structurally fold-free MIDIR (2.01±0.12; Supplementary Table S10). Thus, determinant-level supervision yields warps that are not only locally non-folding but also more globally cycle-consistent, despite the absence of an explicit inverse-consistency loss.

Second, because all folding statistics are computed by finite differencing the predicted field, we test sensitivity to the Jacobian discretization (Supplementary Table S11). Across forward versus central differences, interior versus edge-padded masks, and full versus half resolution, the *ranking* of models is invariant: at every scheme HypEReg-TransMorph has a lower non-positive Jacobian ratio and lower SDlogJ than plain TransMorph. Boundary handling is negligible (interior vs. padded differ by <4% relative for both models), and central differencing slightly lowers all folding counts (HypEReg: $3.39\times 10^{-5}\to 1.51\times 10^{-5}$; TransMorph: $1.88\times 10^{-2}\to 1.55\times 10^{-2}$), so the forward-difference values reported in the main tables are mildly conservative. The one consequential factor is resolution: Evaluating the Jacobian on a half-resolution grid inflates every model’s folding ratio and SDlogJ by roughly one to three orders of magnitude (e.g., HypEReg $3.39\times 10^{-5}\to 4.11\times 10^{-2}$; TransMorph $1.88\times 10^{-2}\to 8.31\times 10^{-2}$), which is why all reported determinant statistics are computed at full field resolution with a single shared implementation across models. The HypEReg-over-TransMorph advantage persists at half resolution as well, so the qualitative conclusion is discretization-robust even though absolute magnitudes are scale-dependent.

## 4. Discussion

### 4.1. Primary Findings

This work is framed as an improvement in deformation reliability rather than a narrow pursuit of overlap alone. Across IXI, HypEReg-TransMorph reduces non-positive Jacobian ratio and SDlogJ while preserving or improving overlap and surface metrics against strong Transformer baselines. Mechanistically, this trade-off matters for any Jacobian-based downstream analysis: deformation fields with high Dice but non-negligible folding report negative or undefined local volume change, which is problematic for tensor-based morphometry, longitudinal change analysis, and label propagation. We deliberately avoid claiming a demonstrated clinical benefit from this property alone; the evidence we provide is methodological reliability plus the targeted morphometry analyses below. The small overlap difference relative to TransMorph and TransMorphBayes (grouped Dice 0.7537 vs. 0.7527/0.7530) should accordingly be read as evidence of *non-inferiority* on overlap—confirmed to lie within multi-seed run-to-run variability (Supplementary Table S12)—rather than as the headline result; the substantive and reproducible effect of HypEReg is the orders-of-magnitude reduction in folding and the tightening of the log-Jacobian distribution at the same overlap, which is precisely the property that makes a deformation field usable for Jacobian-based morphometry.

The downstream multi-atlas experiment and Jacobian morphometry validation (Section 3.7 and Section 3.8) support this interpretation: HypEReg attains the highest fused Dice in zero-shot OASIS transfer (Table 7), shows lower longitudinal disagreement with native nWBV on OASIS-2 (Table 8), and improves ROI-level Jacobian consistency with FastSurfer in the most atrophy-sensitive structures, especially lateral ventricles (Table 9).

### 4.2. Potential Clinical Implications

We outline the clinical relevance of HypEReg as motivation and hypothesis rather than as validated outcomes. Jacobian-derived volume change is the substrate of tensor-based morphometry and of longitudinal atrophy/expansion biomarkers used in Alzheimer’s disease, mild cognitive impairment, multiple sclerosis, normal-pressure hydrocephalus, and post-surgical follow-up. In such pipelines a single folded voxel produces a non-positive or undefined local volume ratio that propagates into voxel-wise statistical maps and ROI-level rates; suppressing folding is therefore a necessary condition for trustworthy Jacobian readouts, not a sufficient one for improved diagnosis. Our updated multi-seed zero-shot OASIS-2 results are consistent with this framing: HypEReg delivers a robust regularity gain (substantially lower non-positive Jacobian ratio and SDlogJ), while clinical-covariate associations remain weak and between-model CDR interaction is not significant. What we have *not* shown—and what would be required before any clinical-utility claim—is a prospective demonstration that fold-suppressed warps change diagnostic sensitivity, biomarker effect sizes, or reader decisions on a powered clinical cohort. We accordingly position HypEReg as a reliability-enhancing building block whose clinical value is a testable hypothesis for future task-level studies (Section 4.6). We further stress that statistical significance and clinical significance are distinct: paired inferential gains on deformation-regularity metrics do not by themselves establish clinical utility. Whether the orders-of-magnitude folding reduction translates into clinically significant changes can only be answered by powered, task-level outcome studies on patient cohorts.

### 4.3. Comparison with MIDIR: Accuracy, Generalization, and Efficiency

MIDIR is a particularly strong comparator because it guarantees fold-free transforms through B-spline parameterization and is computationally efficient. In our IXI evaluation, however, HypEReg-TransMorph remains superior in accuracy-related endpoints: grouped Dice is higher (0.7537 vs. 0.7423), ASSD is lower with significant paired differences, and HD95 is statistically comparable. This pattern indicates that architectural fold prevention alone does not guarantee best anatomical correspondence.

The gap widens under strict IXI→OASIS transfer. HypEReg-TransMorph maintains a larger advantage in Dice and surface metrics, suggesting that loss-side Jacobian supervision combined with dense-flow Transformer expressivity adapts better to cohort shift than a fixed-grid spline parameterization. MIDIR still offers a real efficiency advantage (lower latency and much smaller parameter count), so the practical choice is deployment-dependent: MIDIR is attractive under hard compute constraints, whereas HypEReg-TransMorph is preferable when cross-domain fidelity and fusion quality are priority targets.

### 4.4. Mechanistic Positioning and Portability

HypEReg’s main engineering advantage is that it acts on the training objective, not the inference graph. Fold suppression is learned through explicit determinant-level penalties, while runtime architecture, parameter count, and feed-forward speed remain unchanged relative to the underlying TransMorph backbone. This makes HypEReg portable to other dense-flow Transformer designs without re-architecting deployment pipelines.

Conceptually, this is complementary to methods that emphasize different structural constraints, including inverse-consistency objectives (ICON/GradICON), hierarchical composition (LapIRN), velocity-field integration (TransMorph-Diff/TVF) [7,16,17], and spatially varying regularization (TM-SPR) [18]. Those approaches enforce invertibility structurally or through integration; HypEReg instead applies a soft determinant-level penalty on a direct displacement field. Combining these ideas is therefore plausible rather than contradictory.

### 4.5. Relation to Prior Hyperelastic Regularization

Relative to prior hyperelastic-style regularizers, HypEReg is distinguished by operating directly at the Jacobian determinant level with a coupled design: a clamped-rational volume-distortion term plus an explicit anti-folding hinge. The selective Jacobian penalty in Mok and Chung [10] is closest to the hinge component, but does not provide the same non-linear stabilization away from folding regimes. BMR-inspired volume-change/curvature regularization [11] and conformal-invariant energy approaches [12] are important precedents, mostly demonstrated in CNN-dominant lung CT contexts. To state the nature of the contribution explicitly: HypEReg is a *reformulation* of the BMR hyperelastic family for deep stochastic optimization, not a wholly new energy class. Its novelty is not the use of a hyperelastic prior per se—which is well established—but (i) the specific clamped-rational volume term ${\left(\det J_{\phi }-1\right)}^{2}/\max\left(\det J_{\phi },\epsilon \right)$ that replaces the unbounded BMR quartic with a form yielding finite, well-conditioned gradients under SGD while retaining the compressive/expansive asymmetry, (ii) its explicit coupling with a differentiable anti-folding hinge so that folded voxels are penalized by both terms simultaneously, and (iii) its formulation as a purely loss-side, determinant-level module that leaves the inference graph of a Transformer dense-flow backbone unchanged. Our empirical contribution is to show that this combination suppresses folding by orders of magnitude at non-inferior overlap, with subject-level paired inference, on both IXI and zero-shot OASIS.

### 4.6. Limitations and Future Work

Several limitations remain. First, the headline IXI comparison in Table 2 reports single-run checkpoints per method; to address run-to-run variability we additionally retrained TransMorph and HypEReg-TransMorph under three seeds and confirmed that training-seed variability is small relative to the regularity gains (Supplementary Table S12), but a larger multi-seed budget covering every baseline (e.g., TransMorphBayes) is still outstanding. Second, the strict zero-shot IXI→OASIS *overlap/surface* evaluation uses only the released n=19 Learn2Reg test pairs, which limits precision for cross-cohort overlap metrics. We retain this benchmark because it is the standard, citable OASIS cross-subject protocol, but we do not over-interpret the small-sample Dice/HD95/ASSD differences. The *regularity* conclusion, by contrast, is supported at much larger scale: a complementary large-sample analysis on 393 consecutive labeled OASIS cross-subject pairs reproduces the per-ROI folding/SDlogJ advantage of HypEReg-TransMorph under the same zero-shot transfer (all six paired contrasts $q<10^{-60}$; Supplementary Table S14), and the OASIS-2 longitudinal analysis adds a further 223 within-subject visit pairs from 150 subjects with ROI-integrated Jacobian checks against FastSurfer rates. ROI-level clinical interaction effects nonetheless remain weak and non-significant after correction, so biomarker-level claims remain premature; large-*n* cross-cohort *overlap* validation on a curated multi-site test set is still outstanding. Third, HypEReg offers no hard topological guarantee: it is a training-time penalty, so invertibility is enforced empirically (low but non-zero folding, low inverse-consistency error) rather than by construction as in velocity-field integration or B-spline parameterizations. Fourth, although strict zero-shot transfer and OASIS-retrained supplementary analyses provide cross-cohort evidence, broader pathology-rich external validation and a prospective clinical-endpoint study remain necessary before any diagnostic-utility claim.

Future work should prioritize (i) extending multi-seed retraining to all baselines with full uncertainty decomposition, (ii) additional external cohorts and multimodal protocols, (iii) hybrid formulations that couple HypEReg with inverse-consistent or hierarchical objectives, and (iv) task-level clinical studies quantifying how fold suppression changes biomarker sensitivity, diagnostic confidence, and workflow time.

## 5. Conclusions

We have introduced HypEReg, a non-linear hyperelastic regularizer that couples a clamped-rational volume-distortion term with an explicit anti-folding hinge and acts directly on the Jacobian determinant of the predicted displacement field. Integrated into a TransMorph dense-flow backbone, the regularizer produces HypEReg-TransMorph, a learned brain MRI registration model that delivers near-diffeomorphic deformations at zero inference cost. On the IXI atlas-to-subject benchmark with 115 held-out test subjects, HypEReg-TransMorph reduces the non-positive Jacobian-determinant ratio by approximately three orders of magnitude relative to TransMorph and TransMorphBayes (1.502% → 0.0015%), reduces SDlogJ from 0.5064 to 0.3280, improves grouped Dice over TransMorph (0.7537 vs. 0.7527) and significantly reduces ASSD (1.357 mm vs. 1.407 mm for TransMorph), while adding no inference latency or parameter overhead (Table 5). A strict IXI→OASIS zero-shot evaluation confirms that HypEReg-TransMorph attains the highest Dice and the lowest non-positive Jacobian ratio in the zero-shot group—roughly two orders of magnitude below plain TransMorph zero-shot (Section 3.4; Table 4; Figure 3). The regularity advantage extends to downstream clinical-style pipelines insofar as multi-atlas label fusion on OASIS (Section 3.7; Table 7) shows improved fused overlap when folding is suppressed, consistent with more consistent per-atlas label propagation.

These results address a long-standing usability gap of learning-based registration: Jacobian-based morphometry and atlas-fusion pipelines assume one-to-one, fold-free warps, an assumption that unconstrained Transformer backbones routinely violate. HypEReg-TransMorph improves the *reliability* of Jacobian-based readouts—reducing folding and stabilizing log-Jacobian statistics—as supported by OASIS-2 longitudinal nWBV consistency, OASIS-2 clinical-covariate analyses, and ROI-level agreement with FastSurfer (especially ventricular expansion); it should not be interpreted as having validated new clinical biomarkers without task-specific outcome studies. By delivering near-diffeomorphic fields at feed-forward inference speed and without changes to the deployment architecture, HypEReg-TransMorph is a practical drop-in upgrade for Transformer-based deformable brain MRI registration.

## Figures and Tables

**Table 1:** Dataset, preprocessing, and protocol summary.

Item	Configuration
Dataset source	IXI project dataset [20] and preprocessed IXI package used in TransMorph-style workflows
License	IXI public dataset terms (CC BY-SA 3.0 at IXI portal); preprocessed release follows its repository terms
Volumes and split	576 preprocessed subjects + atlas; 403/58/115 (train/validation/test)
Modality and task	T1-weighted structural MRI; atlas-to-subject deformable registration
Input shape	160×192×224 after preprocessing/cropping
Preprocessing summary	Public preprocessed IXI workflow (including skull stripping, affine alignment, and segmentation preprocessing) with template-space normalization; same pipeline used across all compared methods
Labels and grouped VOIs	Segmentation labels are aggregated into 17 bilateral/related VOI groups for grouped Dice analysis
Atlas definition	Fixed atlas image/label pair; same atlas used for all methods
Interpolation	Intensity: trilinear warping; labels: nearest-neighbor warping
Metric spacing	Surface metrics (HD95/ASSD in the main tables; NSD@1 mm for a model subset in Supplementary Table S2) use spacing-aware distance transforms in the evaluation implementation
Compared families	TransMorph-family baselines, CNN/hybrid baselines, and classical SyN
*OASIS cohort (Section 3.4, Section 3.5, Section 3.6 and Section 3.7; supplementary retraining Table S6)*	
Dataset and benchmark definition	Open Access Series of Imaging Studies (OASIS) [21]; Learn2Reg challenge variant, splits, and preprocessing for cross-subject registration [22]
Public availability	Primary imaging distributed through the OASIS portal (https://www.oasis-brains.org/ (accessed on 11 April 2026)); usage terms per [21]. Challenge materials and dataset documentation [22].

**Table 2:** Main IXI model comparison (115-subject protocol).

Model	Dice ↑	$\det(J_{\phi })\leq 0$ Ratio ↓	SDlogJ ↓	HD95 (mm) ↓	ASSD (mm) ↓
HypEReg-TransMorph	**0.7537 ± 0.0275**	0.000015 ± 0.000007	0.3280 ± 0.0221	$\underline{5.3234\pm 0.6936}$	**1.3570 ± 0.1770**
TransMorph	0.7527 ± 0.0305	0.015021 ± 0.003416	0.5064 ± 0.0250	5.6872 ± 0.7810	$\underline{1.4073\pm 0.1819}$
TransMorphBayes	$\underline{0.7530\pm 0.0302}$	0.015634 ± 0.003363	0.4920 ± 0.0330	5.7246 ± 0.7604	1.4160 ± 0.1832
FL TransMorph-Diff	0.5943 ± 0.0455	**0.000000 ± 0.000000**	**0.0048 ± 0.0003**	8.6391 ± 1.0670	2.4632 ± 0.3380
FL VoxelMorph-1	0.7293 ± 0.0290	0.015860 ± 0.003388	0.4999 ± 0.0327	5.7156 ± 0.8168	1.4696 ± 0.1962
CycleMorph	0.7366 ± 0.0303	0.017192 ± 0.003819	0.5176 ± 0.0320	5.7789 ± 0.8317	1.4638 ± 0.2038
MIDIR	0.7423 ± 0.0228	**0.000000 ± 0.000000**	0.3148 ± 0.0242	**5.3028 ± 0.6139**	1.4100 ± 0.1585
CoTr	0.7347 ± 0.0290	0.012975 ± 0.003430	0.4874 ± 0.0331	5.5691 ± 0.7931	1.4278 ± 0.1828
nnFormer	0.7472 ± 0.0294	0.015946 ± 0.003584	0.5167 ± 0.0371	5.8274 ± 0.8195	1.4322 ± 0.1878
PVT	0.7273 ± 0.0330	0.018578 ± 0.003141	0.5431 ± 0.0318	5.9286 ± 0.8264	1.5253 ± 0.2069
SyN (ANTs)	0.6445 ± 0.0397	$\underline{0.000001\pm 0.000005}$	FL $\underline{0.2996\pm 0.0725}$ FL	6.4457 ± 1.8104	1.5233 ± 0.7748

Values are reported as mean ± standard deviation over 115 test subjects. Arrow keys: ↑ higher is better; ↓ lower is better. Dice is the grouped mean over 17 anatomical structure groups; $\det(J_{\phi })\leq 0$ ratio, SDlogJ, HD95, and ASSD are taken from per-case evaluation exports. **Bold red** indicates the best value per metric; $\underline{\mathrm{blue underline}}$ indicates the second-best. TransMorph-Diff is a diffeomorphic, velocity-field-integration variant of the TransMorph backbone (scaling-and-squaring of a stationary velocity field); it is structurally fold-free ($\det(J_{\phi })\leq 0$ ratio = 0) and attains the lowest SDlogJ, but at a substantial cost in overlap and surface accuracy (lowest Dice, highest HD95/ASSD), illustrating that regularity metrics must be read jointly with accuracy rather than in isolation.

**Table 3:** Ablation study: term-isolation and FL full coefficient-grid FL runs on the FL 58-subject FL IXI FL *validation* split (the same split used for operating-point selection; no test-set feedback)FL. All runs use the same TransMorph backbone and training schedule. β=0 removes $L_{\mathrm{volume}}$; γ=0 removes $L_{\mathrm{fold}}$. The row marked (†) is the FL pre-specified FL operating pointFL at $\epsilon =10^{-3}$; two additional rows vary $\epsilon \in \{10^{-4},10^{-2}\}$ at fixed (β,γ)=(0.02,20)FL. HD95 and ASSD FL use FL the grouped binary-mask method (17 VOI groups, union of constituent labels), consistent with the Dice groupingFL, so FL absolute values differ from the per-label method in Table 2FL; validation Dice is likewise not directly comparable to the held-out test Dice in Table 2FL.

Configuration	β	γ	ϵ	Dice ↑	$\det(J_{\phi })\leq 0$ Ratio ↓	SDlogJ ↓	HD95 ↓	ASSD ↓
Fold hinge only (no $L_{\mathrm{volume}}$)	0.00	20	$10^{-3}$	0.7486 ± 0.0274	$4.49\times 10^{-3}\pm 1.44\times 10^{-3}$	0.4698 ± 0.0382	3.270 ± 0.605	0.769 ± 0.121
Volume term only (no $L_{\mathrm{fold}}$)	0.02	0	$10^{-3}$	0.7497 ± 0.0272	$1.97\times 10^{-5}\pm 7.60\times 10^{-6}$	0.3337 ± 0.0215	3.126 ± 0.477	0.756 ± 0.113
Combined	0.01	10	$10^{-3}$	0.7518 ± 0.0248	$0.55\times 10^{-4}\pm 0.19\times 10^{-4}$	0.3532 ± 0.0234	3.091 ± 0.463	0.748 ± 0.104
Combined	0.01	20	$10^{-3}$	0.7510 ± 0.0261	$4.68\times 10^{-5}\pm 1.40\times 10^{-5}$	0.3523 ± 0.0239	3.109 ± 0.494	0.751 ± 0.110
Combined	0.01	50	$10^{-3}$	0.7504 ± 0.0260	$5.75\times 10^{-5}\pm 1.66\times 10^{-5}$	0.3481 ± 0.0223	3.063 ± 0.456	0.751 ± 0.107
Combined	0.02	10	$10^{-3}$	0.7496 ± 0.0257	$1.98\times 10^{-5}\pm 6.56\times 10^{-6}$	0.3319 ± 0.0207	3.052 ± 0.466	0.753 ± 0.108
Combined, operating point ^†^	0.02	20	$10^{-3}$	0.7510 ± 0.0267	$1.59\times 10^{-5}\pm 6.11\times 10^{-6}$	0.3267 ± 0.0219	3.114 ± 0.497	0.750 ± 0.113
Combined	0.02	50	$10^{-3}$	0.7504 ± 0.0282	$2.06\times 10^{-5}\pm 0.78\times 10^{-5}$	0.3274 ± 0.0207	3.045 ± 0.464	0.751 ± 0.116
Combined (ϵ-sweep)	0.02	20	$10^{-4}$	0.6785 ± 0.0380	$1.73\times 10^{-6}\pm 1.87\times 10^{-6}$	0.3034 ± 0.0127	3.706 ± 0.569	1.031 ± 0.182
Combined (ϵ-sweep)	0.02	20	$10^{-2}$	**0.7526 ± 0.0262**	$1.69\times 10^{-4}\pm 0.40\times 10^{-4}$	0.3440 ± 0.0215	3.048 ± 0.484	**0.742 ± 0.110**
Combined	0.05	10	$10^{-3}$	0.7484 ± 0.0256	$0.48\times 10^{-5}\pm 0.31\times 10^{-5}$	**0.2906 ± 0.0180**	3.073 ± 0.442	0.757 ± 0.107
Combined	0.05	20	$10^{-3}$	0.7514 ± 0.0254	$0.58\times 10^{-5}\pm 0.52\times 10^{-5}$	$\underline{0.2911\pm 0.0173}$	**2.999 ± 0.413**	$\underline{0.744\pm 0.106}$
Combined	0.05	50	$10^{-3}$	0.7487 ± 0.0259	$\underline{0.46\times 10^{-5}\pm 0.35\times 10^{-5}}$	0.2947 ± 0.0166	$\underline{3.020\pm 0.421}$	0.755 ± 0.108

Values: mean ± std over 58 IXI validation subjects. **Bold red** = best per metric; $\underline{\mathrm{blue underline}}$ = second-best. Arrow keys: ↑ higher is better; ↓ lower is better. These are validation-split numbers used for model selection and are therefore distinct from the held-out test results in Table 2 (the operating point’s test Dice is 0.7537). Grouped Dice across the regularized (β,γ) cells spans only 0.7484–0.7518 (within one cross-subject standard deviation), evidencing a broad overlap optimum. The moderate, pre-specified operating point (0.02,20) is deliberately retained (see text), so the reported test results are a conservative operating choice.

**Table 4:** IXI→OASIS zero-shot cross-cohort generalization (mean ± std over 19 OASIS test pairs, n=19). All learned models use IXI-trained checkpoints evaluated on OASIS without any fine-tuning. Classical SyN is shown as a reference at the bottom.

Model	Training	Dice ↑	$\det(J_{\phi })\leq 0$ Ratio ↓	SDlogJ ↓	HD95 (mm) ↓	ASSD (mm) ↓
*IXI-trained zero-shot transfer (no OASIS fine-tuning)*						
HypEReg-TransMorph	IXI	**0.7756 ± 0.0300**	$\underline{7.6\times 10^{-5}\pm 3.9\times 10^{-5}}$	$\underline{3.3085\pm 0.0109}$	**2.4828 ± 0.5253**	**0.7515 ± 0.1080**
TransMorph	IXI	$\underline{0.7691\pm 0.0314}$	0.0096 ± 0.0019	0.4703 ± 0.0197	$\underline{2.6267\pm 0.5207}$	$\underline{0.7832\pm 0.1118}$
TransMorphBayes	IXI	0.7587 ± 0.0352	0.0089 ± 0.0019	0.4593 ± 0.0186	2.7522 ± 0.5553	0.8188 ± 0.1194
MIDIR	IXI	0.7254 ± 0.0291	**0.0000 ± 0.0000**	**0.2551 ± 0.0123**	2.8926 ± 0.4944	0.9306 ± 0.1079
CycleMorph	IXI	0.7243 ± 0.0388	0.0082 ± 0.0011	0.4345 ± 0.0165	3.0759 ± 0.5840	0.9485 ± 0.1380
VoxelMorph-1	IXI	0.7159 ± 0.0477	0.0081 ± 0.0013	0.4291 ± 0.0177	3.1806 ± 0.6656	0.9632 ± 0.1684
PVT	IXI	0.6360 ± 0.0374	0.0162 ± 0.0012	0.5304 ± 0.0111	3.9693 ± 0.6765	1.2673 ± 0.1841
*Classical reference*						
SyN (ANTs)	iterative	0.7385 ± 0.0498	$2.2\times 10^{-7}$ ± $9.4\times 10^{-7}$	0.2075 ± 0.0360	2.7925 ± 0.6341	0.9112 ± 0.2014

Values: mean ± std over 19 OASIS test pairs. Arrow keys: ↑ higher is better; ↓ lower is better. Within the zero-shot group: **Bold red** = best, $\underline{\mathrm{blue underline}}$ = second-best per metric. MIDIR achieves zero non-positive Jacobian ratio via B-spline parameterization (structurally enforced, independent of domain); it is bolded for non-positive Jacobian ratio among learned methods. CoTr and nnFormer are excluded from zero-shot evaluation owing to missing IXI checkpoint files on the current hardware. All zero-shot adapters load the identical IXI validation checkpoints used for Table 2; no OASIS examples are seen during training.

**Table 5:** Pure forward-pass profiling comparison (runtime, peak memory, and parameter count) for learned baselines.

Model	Runtime (s/Case) ↓	Peak Memory (GB) ↓	Parameters (M)
HypEReg-TransMorph	0.0822	5.685	46.771
TransMorphBayes	0.0852	6.042	46.773
TransMorph	0.0822	5.685	46.771
VoxelMorph-1	0.0264	4.467	0.274
CycleMorph	0.0418	2.973	0.361
MIDIR	0.0149	1.994	0.266
CoTr	0.1023	5.951	38.738
nnFormer	0.0533	2.559	45.328
PVT	0.1452	4.944	61.841

Arrow keys: ↓ lower is better. Values are measured by no-gradient pure forward profiling on synthetic inputs of size 1×2×160×192×224, with warm-up plus repeated timing runs, and peak CUDA memory. Classical SyN (ANTs) is omitted because iterative CPU optimization is not comparable to a single GPU forward pass under this protocol. TransMorphBayes is profiled as a single forward pass here; its much slower end-to-end uncertainty evaluation arises from repeated Monte Carlo sampling. Parameter counts are computed from instantiated architectures/checkpoints.

**Table 6:** Training cost of parametric deep-learning registration models: a single forward–backward optimization step on a 1×2×160×192×224 input. FLOPs count convolution, matmul and attention operations via PyTorch’s operator-level FLOP counter (elementwise and grid-sample operations are not counted); parameters, FLOPs and peak memory are deterministic, whereas the per-step wall time is an indicative mean on one shared RTX PRO 6000. Classical SyN/Affine are excluded (no trainable parameters).

Model	Params (M)	Fwd GFLOPs	Train GFLOPs (f+b)	Step (ms)	Peak Mem (GB)
HypEReg-TransMorph	46.771	1447.2	4322.7	253.7	12.23
TransMorph	46.771	1447.2	4322.7	240.4	12.23
TransMorphBayes	46.773	1447.2	4322.7	246.1	13.47
TransMorph-Diff	46.557	637.2	1904.4	131.9	6.08
VoxelMorph-1	0.274	608.1	1823.6	102.7	5.39
CycleMorph	0.361	252.9	746.7	87.9	3.77
MIDIR	0.266	94.3	270.9	42.8	3.08
CoTr	38.738	4315.8	12,796.4	308.2	15.87
nnFormer	45.328	314.8	941.7	144.9	8.23
PVT	61.841	519.3	1543.9	534.3	10.55

Single-step training profiling (3 warm-up + 6 timed steps) with the Adam(AMSGrad) optimizer. HypEReg-TransMorph additionally evaluates the hyperelastic loss in its step; it shares the TransMorph backbone, so its parameters and FLOPs are identical to TransMorph and the regularizer’s cost appears only as a small wall-time/memory increment (see text). FLOPs are reported for countable convolution/matmul/attention operations; cheap elementwise and grid-sample operations (including the HypEReg Jacobian penalties) are not included, which is why the HypEReg and TransMorph FLOP columns coincide.

**Table 7:** Multi-atlas label fusion on OASIS zero-shot transfer ($N_{\mathrm{atlas}}=6$, n=20 test targets). Majority-voting fusion of six atlas-to-target registrations; $\Delta \mathrm{Dice}={\mathrm{Dice}}_{\mathrm{fusion}}-{\bar{\mathrm{Dice}}}_{\mathrm{single}}$.

Model	Single Dice ↑	Fused Dice ↑	ΔDice ↑	Hippocampus ↑	Ventricle ↑	Thalamus ↑
HypEReg-TransMorph	**0.7795 ± 0.0159**	**0.8271 ± 0.0181**	+0.0477	**0.8654**	**0.9056**	**0.9227**
TransMorph	$\underline{0.7712\pm 0.0146}$	$\underline{0.8201\pm 0.0145}$	$\underline{+0.0489}$	$\underline{0.8492}$	$\underline{0.9006}$	$\underline{0.8979}$
TransMorphBayes	0.7597 ± 0.0185	0.8058 ± 0.0206	+0.0460	0.8341	0.8975	0.8846
MIDIR	0.7161 ± 0.0141	0.7696 ± 0.0162	**+0.0534**	0.8238	0.8719	0.8955

Arrow keys: ↑ higher is better. Values: mean ± std over 20 OASIS test targets. $N_{\mathrm{atlas}}=6$ atlas subjects (IDs: 50, 80, 150, 220, 300, 380; ID 80 substitutes for the originally planned ID 100, which is absent in the release split). Single Dice = per-atlas mean; Fused Dice = majority-voting result. **Bold red** = best; $\underline{\mathrm{blue underline}}$ = second-best.

**Table 8:** OASIS-2 longitudinal deformation regularity under IXI→OASIS-2 zero-shot transfer (pair-level Jacobian metrics over n=223 visit intervals; 3 seeds per model family).

Model	$\det(J_{\phi })\leq 0$ Ratio ↓	SDlogJ ↓
HypEReg-TransMorph	** $1.37\times 10^{-3}\pm 8.20\times 10^{-4}$ **	**0.401 ± 0.041**
TransMorph	$2.11\times 10^{-2}\pm 3.45\times 10^{-3}$	0.601 ± 0.015

Across-seed mean ± std from three IXI-trained zero-shot checkpoints per family. Arrow keys: ↓ lower is better. The coarse native ΔnWBV/year sanity check formerly paired with this table is reported in Supplementary Table S15, and the full robustness panel is in Supplementary Table S8. **Bold red** = better, red = Best.

**Table 9:** Numerical summary corresponding to Figure 6. The table is restricted to the three ROI readouts retained in the main text: lateral ventricles, thalamus, and white matter. The left pair of columns reports across-seed Pearson correlations between ROI-integrated Jacobian relative change and FastSurfer-derived ROI volume-change rates. The right pair reports across-seed slopes of ROI Jacobian change versus CDR. Values are mean ± std over three IXI-trained zero-shot checkpoints per model family.

ROI	FS Corr. HypEReg	FS Corr. TransMorph	CDR Slope HypEReg	CDR Slope TransMorph
Lateral ventricles	**0.315 ± 0.045**	0.241 ± 0.042	0.0263 ± 0.0086	0.0184 ± 0.0039
Thalamus	**0.187 ± 0.087**	0.051 ± 0.097	0.0336 ± 0.0027	0.0375 ± 0.0125
White matter	**0.124 ± 0.051**	0.095 ± 0.034	0.0044 ± 0.0029	0.0087 ± 0.0008

FS corr. = Pearson correlation with FastSurfer-derived ROI annualized volume-change rates. CDR slope is the linear slope of ROI-integrated Jacobian relative change versus CDR. Bold indicates the stronger absolute FastSurfer agreement within each ROI; CDR slopes are descriptive and are not interpreted as corrected clinical endpoint validation. **Bold red** = better.

## Data Availability

The data presented in this study are available in IXI at https://brain-development.org/ixi-dataset/(CC BY-SA 3.0) (accessed on 6 April 2026) and the preprocessed split follows the public TransMorph-style IXI preprocessing release. OASIS/OASIS-2 imaging data are available through the Open Access Series of Imaging Studies portal (https://www.oasis-brains.org/) (accessed on 11 April 2026) under the corresponding OASIS data-use terms; the OASIS cross-subject benchmark definitions and splits follow the Learn2Reg challenge materials [22]. Source code, configuration files, per-case metric tables, and figure/statistics scripts are openly available at https://github.com/Zzxmh/HypEReg-TransMorph (accessed on 16 April 2026) and archived at Zenodo: https://doi.org/10.5281/zenodo.19888526 [43]. Trained model weights are available from the corresponding author upon reasonable request.

## Supplementary Materials

The following supporting information can be downloaded at: https://www.mdpi.com/article/10.3390/jimaging12070276/s1, Figure S1, descriptive IXI multi-metric bar-chart overview; Figure S2, qualitative registration comparisons across axial/coronal/sagittal views; Table S1, IXI baseline checkpoint provenance; Table S2, auxiliary IXI deformation/surface metrics (including NSD@1 mm); Table S3, IXI paired inferential summary (mean ± std and aligned median tests); Table S4, IXI paired Wilcoxon/BH-FDR results with effect sizes; Table S5, grouped-VOI label mapping; Table S6, OASIS-retrained paired Wilcoxon/BH-FDR results (distinct from main-text zero-shot); Table S7, bootstrap 95% confidence intervals on IXI subject-level means; Table S8, OASIS-2 longitudinal Jacobian–nWBV consistency (full panel); Table S9, OASIS-2 clinical association tests; Table S10, inverse-consistency error on IXI; Table S11, Jacobian discretization sensitivity (forward/central, interior/padded, full/half resolution); Table S12, per-seed and across-seed training variability on IXI for TransMorph and HypEReg-TransMorph; Table S13, large-sample (n=393) IXI→OASIS zero-shot per-ROI Jacobian plausibility; Table S14, OASIS-2 ROI clinical-pattern summaries; Table S15, compact native ΔnWBV/year sanity summary; Table S16, FastSurfer small-scale sanity validation; Supplementary Text, model/training details and OASIS cross-cohort training environment.

## Abbreviations

The following abbreviations are used in this manuscript:

HypEReg	Hyperelastic-Energy Regularization
MRI	Magnetic Resonance Imaging
HD95	95th percentile Hausdorff Distance
ASSD	Average Symmetric Surface Distance
NCC	Normalized Cross-Correlation
LNCC	Local Normalized Cross-Correlation
SDlogJ	Standard Deviation of Log Jacobian Determinant
IXI	Information eXtraction from Images dataset
BMR	Burger–Modersitzki–Ruthotto (hyperelastic energy framework)
FDR	False Discovery Rate
