# Supplementary material for: Hyperelastic Regularization for Near-Diffeomorphic Transformer-Based Brain MRI Registration
Source: J Imaging. 2026 Jun 24;12(7):276. doi: 10.3390/jimaging12070276 (PMC13413009; doi:10.3390/jimaging12070276)
Supplement: Supplementary file 1 [file jimaging-12-00276-s001.zip › supplementary_diff.pdf]

Supplementary Materials
Hyperelastic Regularization for Near-Diffeomorphic Transformer-Based Brain MRI Registration

This document accompanies the manuscript *Hyperelastic Regularization for Near-Diffeomorphic Transformer-Based Brain MRI Registration*. Contents include training hyperparameters and evaluation notes, IXI baseline checkpoint summary (Table S1), auxiliary IXI deformation/surface metrics omitted from the main comparison table (Table S2), paired inferential exports on IXI (Tables S3–S4 under *Inferential Statistics*), grouped-VOI mapping (Table S5), Figure S1 (descriptive IXI bar-chart overview), Figure S2 (qualitative registration comparison across three orthogonal views), OASIS training notes, OASIS-retrained contrasts versus selected baselines (Table S6), bootstrap 95% confidence intervals on IXI means (Table S7), OASIS-2 longitudinal Jacobian–nWBV consistency (Table S8), OASIS-2 clinical association tests (Table S9), inverse-consistency error on IXI (Table S10), Jacobian discretization sensitivity (Table S11), multi-seed training variability on IXI (Table S12), large-sample (n=393) IXI→OASIS zero-shot per-ROI Jacobian plausibility (Table S13), ADNI-style OASIS-2 ROI clinical-pattern summaries (Table S14), compact nWBV sanity summary (Table S15), and FastSurfer small-scale sanity validation (Table S16).

1 Supplementary Experimental Details

1.1 Model and Environment Configuration

The following settings are sufficient to reproduce HypEReg-TransMorph training on the IXI atlas-to-subject protocol with the public TransMorph-style backbone.

Software. Python 3.12.x; PyTorch (CUDA build matched to the GPU). Classical baselines and distance transforms use antspyx and SimpleITK as in the main evaluation. The reported experiments used PyTorch 2.12 (CUDA 12.8) on one NVIDIA RTX PRO 6000 (97 GiB VRAM); other CUDA-capable GPUs are suitable (reduce batch size if memory-limited).

Data protocol. Preprocessed IXI T1 volumes in template space, spatial size $160 \times 192 \times 224$, with the standard 403/58/115 train/validation/test split and a single fixed atlas. Training batch size 2; validation batch size 1. Training augmentations: independent random flips along each spatial axis with probability 0.5; cast image pairs to float32. Validation: FreeSurfer-compatible label relabeling to the 46-label index set used in this study, then float32 images and int16 labels.

Optimization. Adam with AMSGrad, learning rate 10^{-4}, weight decay 0, and PyTorch defaults $(\beta_1, \beta_2, \epsilon) = (0.9, 0.999, 10^{-8})$. Train for 500 epochs with bidirectional updates ($x \to y$ and $y \to x$): one forward–backward–optimizer step per direction per mini-batch. Learning-rate decay each iteration:

$$\mathrm{lr}(e) = \mathrm{lr}_0 \left(1 - \frac{e}{500}\right)^{0.9}.$$

Retain the checkpoint with the best validation grouped Dice. As in the upstream TransMorph reference script, a single global random seed is not pinned; the released weights define the reference run (see main-text limitations on multi-seed retraining).

Loss. Total loss per direction (unit weights on all three terms):

$$\mathcal{L}_{\text{total}} = \mathcal{L}_{\text{sim}} + \mathcal{L}_{\text{grad}} + \mathcal{L}_{\text{HypEReg}}.$$

Similarity $\mathcal{L}_{\text{sim}} = -\overline{\text{LCC}}^2$ with a 9^3 local window and denominator stabilizer 10^{-5}. Smoothness $\mathcal{L}_{\text{grad}}$: mean squared first-order finite differences of the displacement along x, y, and z, averaged over the three axes. HypEReg weights $(\beta, \gamma, \epsilon) = (0.02, 20, 10^{-3})$; BMR-style length/surface terms are disabled ($\alpha_{\text{length}} = 0$). Jacobian $J_\phi = I + \nabla u$ from forward finite differences on interior voxels; volume term $(\det J_\phi - 1)^2 / \max(\det J_\phi, \epsilon)$; folding term $[\max(0, \epsilon - \det J_\phi)]^2$ (equivalently $\text{ReLU}(\epsilon - \det J_\phi)^2$).

TransMorph backbone. Patch size 4; input channels 2; spatial size $(160, 192, 224)$; embed dimension 96; encoder depths $(2, 2, 4, 2)$; attention heads $(4, 4, 8, 8)$; window sizes $(5, 6, 7, 7)$; MLP ratio 4; patch-merging reduction factor 4; registration-head channels 16; skip connections from Transformer stages and from the encoder enabled; query/key/value bias disabled; dropout 0; stochastic depth rate 0.3; relative position bias enabled; patch normalization on; absolute/relative sinusoidal PE off; gradient checkpointing off; decoder feature indices $(0, 1, 2, 3)$. The released HypEReg-TransMorph and TransMorph checkpoints instantiate $\sim$46.77 M parameters.

Forward runtime profiling (main text). Profile in no-gradient mode, input shape $1 \times 2 \times 160 \times 192 \times 224$, five warm-up iterations and twenty timed repeats; report mean wall time and peak device memory. On the hardware above, HypEReg-TransMorph and TransMorph yield 0.0822 s and 5.685 GB; TransMorphBayes yields 0.0852 s and 6.042 GB.

Table S1: Baseline checkpoints used for IXI evaluation.

**Model**	**Checkpoint / source note**
HypEReg-TransMorph	HypEReg-TransMorph; validation-selected IXI run (HypEReg weights β=0.02, γ=20).
TransMorph	Public TransMorph IXI checkpoint from the paper release (validation DSC $\approx$ 0.744).
TransMorphBayes	Bayesian TransMorph checkpoint from the same release (validation DSC $\approx$ 0.743).
MIDIR	Released MIDIR validation checkpoint (DSC $\approx$ 0.733).
CycleMorph	Released CycleMorph validation checkpoint (DSC $\approx$ 0.729).
VoxelMorph-1	Released single-resolution VoxelMorph checkpoint (DSC $\approx$ 0.720).
CoTr	Released CoTr checkpoint (DSC $\approx$ 0.730).
nnFormer	Released nnFormer checkpoint (DSC $\approx$ 0.739).
PVT	Released PVT checkpoint (DSC $\approx$ 0.720).
SyN (ANTs)	Classical SyN outputs under the shared IXI protocol (no network weights).

2 Descriptive IXI Overview

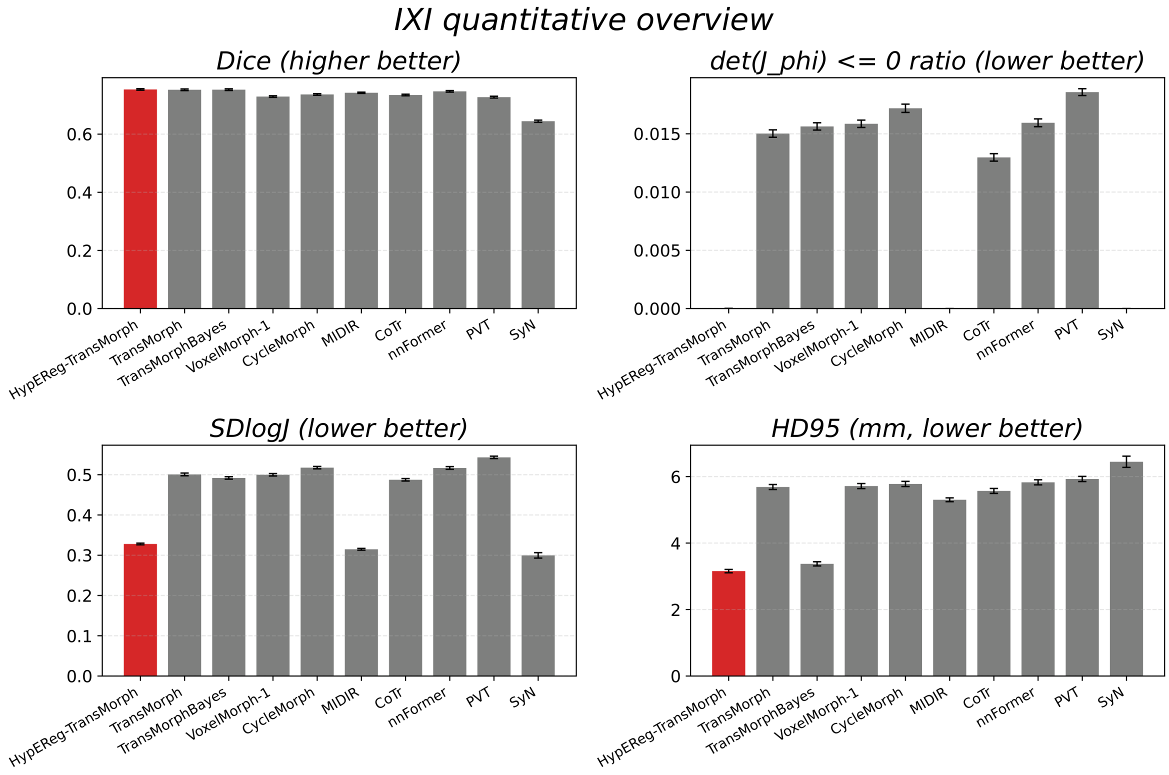

Figure S1: Descriptive IXI quantitative overview (Dice, non-positive Jacobian determinant ratio, SDlogJ, and HD95). Bars include standard-error error bars for visual uncertainty context.

3 Qualitative Registration Comparison

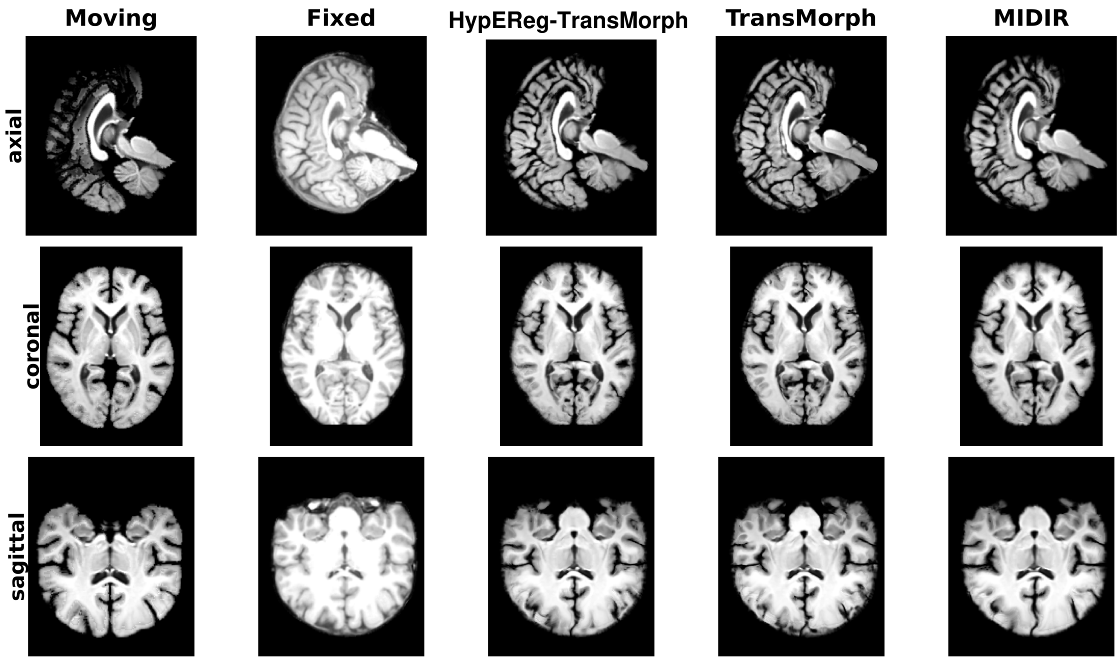

Figure S2: Qualitative registration comparison on a representative IXI test subject across three orthogonal views (axial/coronal/sagittal). Columns show moving atlas, fixed target, and warped outputs from HypEReg-TransMorph, TransMorph, and MIDIR. Boundary-alignment behavior in ventricles, cortex, and deep gray structures complements the deformation-grid and Jacobian evidence presented in the main text. Column titles in the figure panels use the abbreviated name HypEReg-TransMorph for the proposed method.

4 Inferential Statistics (HypEReg-TransMorph vs. Baselines)

Metric definitions (to avoid naming ambiguity). In this Supplementary section, 46-label Dice mean denotes the per-subject mean Dice over the full 46-label FreeSurfer-compatible label set in the per-case metric tables, whereas the main paper reports *grouped Dice* averaged over 17 grouped VOIs (Table 2 in the main text). The two numbers therefore have different scales and are not directly comparable.

Table S2: Auxiliary deformation and surface metrics on IXI (mean±std over subjects). This table reports NSD@1 mm and additional deformation-regularity descriptors that are referenced in the main text but not included in Table 2.

**Model**	**NSD@1 mm↑**	**Bending↓**	**MADiv↓**	$J_{\min}$	J_{p01}	J_{p99}	$J_{\max}$
HypEReg-TransMorph	0.8480 ± 0.0317	0.0031 ± 0.0001	0.1772 ± 0.0114	-0.2700 ± 0.1496	0.2754 ± 0.0268	2.1284 ± 0.0669	18.6155 ± 2.9607
TransMorphBayes	0.8462 ± 0.0347	0.0050 ± 0.0003	0.2341 ± 0.0114	-6.6766 ± 2.2896	-0.0871 ± 0.0555	2.5926 ± 0.0764	46.0976 ± 9.2892
HypEReg (volume only)	0.7715 ± 0.0398	0.0036 ± 0.0001	0.1719 ± 0.0077	-0.1754 ± 0.0993	0.3433 ± 0.0196	2.1054 ± 0.0979	12.3397 ± 2.2249
HypEReg+Grad (volume only)	0.7612 ± 0.0403	0.0019 ± 0.0001	0.1549 ± 0.0065	-0.1014 ± 0.0928	0.3729 ± 0.0187	2.0257 ± 0.0840	8.4640 ± 1.1222
HypEReg+Grad (fold only)	0.7242 ± 0.0424	0.0041 ± 0.0001	0.2367 ± 0.0064	-1.2721 ± 0.3317	0.0776 ± 0.0232	2.5189 ± 0.0782	19.8379 ± 2.8103

Bending = voxel-averaged bending energy; MADiv = mean absolute divergence of the displacement field; NSD@1 mm computed with $\tau = 1$ mm. "HypEReg (volume only)" disables the fold hinge ($\gamma = 0$); "HypEReg+Grad" rows include the standard first-order smoothness term $\mathcal{L}_{\text{grad}}$ and then isolate either the volume component ($\gamma = 0$) or the fold component ($\beta = 0$) of HypEReg. Values are consolidated evaluation exports for the listed models.

Table S3: Inferential summary for TransMorphBayes and MIDIR (mean±std, aligned median difference, Wilcoxon p, Benjamini–Hochberg q, and matched-pairs rank-biserial effect). HypEReg-TransMorph denotes the proposed model. Where 46-label Dice mean appears, values are *not* the main-text grouped Dice over 17 VOIs (Table 2); see table footnote.

Metric	Baseline	HypEReg mean±std	Baseline mean±std	Median diff (aligned)	p/q/effect
dice_mean	TransMorphBayes (uploaded ckpt)	0.5765±0.0301	0.5731±0.0313	0.00280	6.58×10^{-10} / 6.58×10^{-10} / 0.6636
non_jec	TransMorphBayes (uploaded ckpt)	$1.46 \times 10^{-5} \pm 7.37 \times 10^{-6}$	0.01510±0.00329	0.01489	1.31×10^{-20} / 3.28×10^{-20} / 1.0000
SDlogJ	TransMorphBayes (uploaded ckpt)	0.3280±0.0221	0.4934±0.0334	0.16325	1.31×10^{-20} / 3.28×10^{-20} / 1.0000
HD95_mean	TransMorphBayes (uploaded ckpt)	5.3234±0.6936	5.7246±0.7604	0.33877	1.77×10^{-18} / 2.94×10^{-18} / 0.9424
ASSD_mean	TransMorphBayes (uploaded ckpt)	1.3570±0.1770	1.4160±0.1832	0.06143	2.98×10^{-11} / 3.72×10^{-11} / 0.7142
dice_mean	MIDIR	0.5765±0.0301	0.5643±0.0244	0.01166	3.79×10^{-18} / 7.76×10^{-18} / 0.9331
non_jec	MIDIR	$1.46 \times 10^{-5} \pm 7.37 \times 10^{-6}$	0.0000±0.0000	-1.31×10^{-5}	1.31×10^{-20} / 3.69×10^{-20} / -1.0000
SDlogJ	MIDIR	0.3280±0.0221	0.3148±0.0242	-0.01365	4.84×10^{-20} / 1.28×10^{-19} / -0.9850
HD95_mean	MIDIR	5.3234±0.6936	5.3028±0.6139	-0.00855	0.5767 / 0.5898 / -0.0600
ASSD_mean	MIDIR	1.3570±0.1770	1.4100±0.1585	0.06514	3.58×10^{-9} / 4.36×10^{-9} / 0.6342

Aligned median difference is oriented so positive values indicate HypEReg-TransMorph improvement for the given metric direction. The effect is matched-pairs rank-biserial correlation. Pair counts: $n = 115$ for both baselines in this table. **46-label Dice mean:** per-subject mean Dice averaged over the full 46-label evaluation set (same convention as the metric-definition paragraph above). Main-text overlap uses *grouped* Dice (mean over 17 aggregated VOIs). The two scalings differ, so 46-label Dice mean means/std/median contrasts must not be compared numerically to grouped-Dice entries in Table 2; Wilcoxon p and BH q on 46-label Dice mean rows test paired differences on this 46-label convention only.

Table S4: Selected paired comparisons (Wilcoxon signed-rank with Benjamini–Hochberg FDR). Positive signed effect indicates HypEReg-TransMorph improvement under metric-aligned directionality; signed effect is matched-pairs rank-biserial correlation. Rows with 46-label Dice mean follow the 46-label per-subject mean convention (not main-text grouped Dice); see table footnote.

Metric	Baseline	Median paired diff (aligned)	p-value	q-value	Signed effect
non_jec	TransMorph	0.014747	1.31×10^{-20}	3.69×10^{-20}	1.0000
SDlogJ	TransMorph	0.170655	1.31×10^{-20}	3.69×10^{-20}	1.0000
HD95_mean	TransMorph	0.319583	3.77×10^{-17}	7.08×10^{-17}	0.9046
ASSD_mean	TransMorph	0.051747	6.09×10^{-11}	7.83×10^{-11}	0.7028
non_jec	MIDIR	-0.000013	1.31×10^{-20}	3.69×10^{-20}	-1.0000
SDlogJ	MIDIR	-0.013652	4.84×10^{-20}	1.28×10^{-19}	-0.9850
HD95_mean	MIDIR	-0.008554	0.5767	0.5898	-0.0600
ASSD_mean	MIDIR	0.065138	3.58×10^{-9}	4.36×10^{-9}	0.6342
non_jec	TransMorph (uploaded ckpt)	0.014747	1.31×10^{-20}	2.63×10^{-20}	1.0000
dice_mean	TransMorph (uploaded ckpt)	0.002326	6.88×10^{-7}	6.88×10^{-7}	0.5334
non_jec	TransMorphBayes (uploaded ckpt)	0.014714	1.31×10^{-20}	2.63×10^{-20}	1.0000
dice_mean	TransMorphBayes (uploaded ckpt)	0.002706	1.74×10^{-10}	2.32×10^{-10}	0.6858
non_jec	SyN	-0.000012	8.00×10^{-16}	1.24×10^{-15}	-0.8654
SDlogJ	SyN	-0.045599	3.09×10^{-5}	3.31×10^{-5}	-0.4477
HD95_mean	SyN	0.705730	1.31×10^{-7}	1.47×10^{-7}	0.5670
ASSD_mean	SyN	-0.111801	0.6572	0.6572	0.0477

46-label Dice mean rows: paired tests use per-subject mean Dice over 46 labels (export field name), which is on a different absolute scale than grouped Dice in main-text Table 2; do not equate magnitudes across documents. All other metrics match the main evaluation pipeline. Pair count $n = 115$ where full paired exports exist.

Additional uploaded-checkpoint paired results for TransMorph and TransMorphBayes refer to 46-label Dice mean (46-label mean per subject, not grouped Dice) and non-positive Jacobian ratio, included with corresponding subject-level inputs for completeness. Repeated values such as $p = 1.31 \times 10^{-20}$ occur when the two-sided Wilcoxon test reaches the practical floating-point floor under near-complete sign consistency for $n = 115$, and should be interpreted as extremely small p-values.

5 Grouped-VOI Mapping

Table S5: Grouped-VOI mapping used for grouped Dice reporting.

**Grouped VOI**	**FreeSurfer-compatible label IDs**
Brain-Stem	16
Thalamus	10, 49
Cerebellum-Cortex	8, 47
Cerebral-White-Matter	2, 41
Cerebellum-White-Matter	7, 46
Putamen	12, 51
VentralDC	28, 60
Pallidum	13, 52
Caudate	11, 50
Lateral-Ventricle	4, 43
Hippocampus	17, 53
3rd-Ventricle	14
4th-Ventricle	15
Amygdala	18, 54
Cerebral-Cortex	3, 42
CSF	24
choroid-plexus	31, 63

6 OASIS Cross-Cohort Training and Environment

Primary OASIS structural MRI are distributed through the public portal `https://www.oasis-brains.org/` (Open Access Series of Imaging Studies). Train/validation/test splits and preprocessing match the Learn2Reg challenge cross-subject registration setup for OASIS; see the main manuscript Methods for citations and for the definition of the preprocessed release used in our experiments.

OASIS in-cohort retraining mirrors the IXI HypEReg-TransMorph recipe: the same TransMorph backbone as above, Adam with AMSGrad (10^{-4} learning rate, weight decay 0), polynomial LR decay with exponent 0.9 over 500 epochs, bidirectional updates, batch size 2 on the OASIS train split, and HypEReg weights $(\beta, \gamma, \epsilon) = (0.02, 20, 10^{-3})$. Use Python/PyTorch builds compatible with the host GPU and the same antspyx/SimpleITK stack as in the IXI evaluation where classical baselines are compared. The OASIS-retrained model used for Supplementary Table S6 is *not* the strict IXI→OASIS zero-shot checkpoint reported in the main text.

7 OASIS Paired Wilcoxon / BH-FDR

Reference model: OASIS-retrained HypEReg-TransMorph. All pairs n=19. Only significant contrasts ($q_{\mathrm{BH}} < 0.05$) shown; sign of direction: + = HypEReg-TransMorph better. Unless otherwise stated, baselines in this table are also *OASIS in-cohort retrained* models evaluated on the same 19 OASIS test pairs. This table is therefore distinct from the main-text IXI→OASIS zero-shot transfer results.

Table S6: Significant paired Wilcoxon contrasts (BH-FDR, $q < 0.05$) for HypEReg-TransMorph vs baselines on 19 OASIS test pairs.

**Baseline**	**Metric**	**HypEReg mean**	**Baseline mean**	p / q
CycleMorph	dice_mean↑	0.7966	0.7243	3.8×10^{-6} / 3.8×10^{-6}
	non_jec↓	1.0×10^{-5}	8.2×10^{-3}	3.8×10^{-6} / 3.8×10^{-6}
	SDlogJ↓	0.2786	0.4345	3.8×10^{-6} / 3.8×10^{-6}
	HD95_mean↓	2.294	3.076	3.8×10^{-6} / 3.8×10^{-6}
	ASSD_mean↓	0.680	0.949	3.8×10^{-6} / 3.8×10^{-6}
MIDIR	dice_mean↑	0.7966	0.7254	3.8×10^{-6} / 3.8×10^{-6}
	non_jec↓	1.0×10^{-5}	0.0000	1.3×10^{-4} / 1.3×10^{-4} (*HypEReg worse*)
	SDlogJ↓	0.2786	0.2551	3.8×10^{-6} / 3.8×10^{-6} (*HypEReg worse*)
	HD95_mean↓	2.294	2.893	3.8×10^{-6} / 3.8×10^{-6}
	ASSD_mean↓	0.680	0.931	3.8×10^{-6} / 3.8×10^{-6}
TransMorph (dsc0.857)	dice_mean↑	0.7966	0.8610	3.8×10^{-6} / 3.8×10^{-6} (*HypEReg worse*)
	non_jec↓	1.0×10^{-5}	8.1×10^{-3}	3.8×10^{-6} / 3.8×10^{-6}
	SDlogJ↓	0.2786	0.4211	3.8×10^{-6} / 3.8×10^{-6}
	HD95_mean↓	2.294	1.598	3.8×10^{-6} / 3.8×10^{-6} (*HypEReg worse*)
	ASSD_mean↓	0.680	0.483	3.8×10^{-6} / 3.8×10^{-6} (*HypEReg worse*)
VoxelMorph-1	dice_mean↑	0.7966	0.7159	3.8×10^{-6} / 3.8×10^{-6}
	non_jec↓	1.0×10^{-5}	8.1×10^{-3}	3.8×10^{-6} / 3.8×10^{-6}
	SDlogJ↓	0.2786	0.4291	3.8×10^{-6} / 3.8×10^{-6}
	HD95_mean↓	2.294	3.181	3.8×10^{-6} / 3.8×10^{-6}
	ASSD_mean↓	0.680	0.963	3.8×10^{-6} / 3.8×10^{-6}

p: two-sided Wilcoxon signed-rank; q: Benjamini–Hochberg FDR. "HypEReg worse" indicates the signed direction favors the baseline on that metric; these contrasts remain significant, indicating MIDIR/dsc0.857 significantly outperform HypEReg-TransMorph on those regularity/overlap dimensions. Reported non_jec values at or near 10^{-5} reflect the evaluation export precision floor on this OASIS split (with MIDIR exactly 0 by construction under B-spline parameterization), so Wilcoxon significance is driven by paired subject-level ranks rather than by visual separation of rounded means alone. Full numeric summaries are reported in the table.

8 Bootstrap 95% Confidence Intervals (IXI)

Table S7: Bootstrap 95% confidence intervals for IXI subject-level metric means (B = 10,000, sampling with replacement, seed = 0, n = 115 per model).

**Model**	**Dice mean [95% CI]**	**det(J_ϕ) ≤ 0 ratio mean [95% CI]**	**SDlogJ mean [95% CI]**	**HD95 mean [95% CI]**	**ASSD mean [95% CI]**
HypEReg-TransMorph	0.7537 [0.7486, 0.7587]	1.461×10^{-5} [1.332×10^{-5}, 1.604×10^{-5}]	0.3280 [0.3240, 0.3322]	5.3234 [5.1952, 5.4511]	1.3570 [1.3247, 1.3897]
TransMorph	0.7527 [0.7470, 0.7581]	1.502×10^{-2} [1.441×10^{-2}, 1.566×10^{-2}]	0.5064 [0.4943, 0.5076]	5.6872 [5.5391, 5.8376]	1.4073 [1.3735, 1.4429]
TransMorphBayes	0.7530 [0.7474, 0.7584]	1.510×10^{-2} [1.451×10^{-2}, 1.571×10^{-2}]	0.4920 [0.4874, 0.4996]	5.7246 [5.5847, 5.8646]	1.4160 [1.3831, 1.4501]
MIDIR	0.7423 [0.7380, 0.7465]	0 [0, 0]	0.3148 [0.3105, 0.3194]	5.3028 [5.1898, 5.4172]	1.4100 [1.3815, 1.4395]
SyN (ANTs)	0.6445 [0.6372, 0.6518]	5.874×10^{-6} [1.235×10^{-6}, 1.243×10^{-5}]	0.2996 [0.2866, 0.3130]	6.4457 [6.1174, 6.7784]	1.5233 [1.3838, 1.6675]

Dice follows the grouped 17-structure protocol used in the main paper; non-positive Jacobian ratio, SDlogJ, HD95, and ASSD come from the same per-case evaluation pipeline. Bootstrap: B = 10,000, sampling with replacement, seed 0.

9 OASIS-2 Longitudinal Jacobian–nWBV Consistency (Robustness to Aggregation)

Table S8: OASIS-2 longitudinal consistency between the brain-mask Jacobian atrophy proxy and native nWBV change, under five complementary formulations. "rate" uses mean $\log\det J_\phi$/year vs ΔnWBV/year; "total" uses interval mean $\log\det J_\phi$ vs ΔnWBV. The within-subject row demeans each subject's values (removing between-subject variance; subjects with ≥ 2 intervals, $n = 56$ subjects / 129 pairs). CDR>0 restricts to the atrophy subset (105 pairs).

Model	Analysis	n	Pearson r [95% CI]	p	MAE	RMSE
HypEReg-TransMorph	subject-mean (rate)	150	0.014 $[-0.132, 0.180]$	0.862	0.093	0.102
	all pairs (rate)	223	0.055 $[-0.055, 0.171]$	0.414	0.099	0.119
	all pairs (total)	223	0.026 $[-0.098, 0.159]$	0.701	0.167	0.179
	within-subject (rate)	129	-0.006 $[-0.175, 0.221]$	0.942	0.037	0.064
	CDR>0 (rate)	105	0.049 $[-0.078, 0.232]$	0.623	0.105	0.133
TransMorph	subject-mean (rate)	150	0.050 $[-0.091, 0.190]$	0.541	0.212	0.226
	all pairs (rate)	223	0.102 $[-0.007, 0.209]$	0.129	0.220	0.246
	all pairs (total)	223	0.089 $[-0.033, 0.214]$	0.185	0.379	0.395
	within-subject (rate)	129	0.124 $[-0.043, 0.285]$	0.162	0.070	0.100
	CDR>0 (rate)	105	0.143 [0.017,0.281]	0.145	0.224	0.252

Pearson 95% CI from $B = 2000$ bootstrap resamples. *Individual-level linear correlation with nWBV is weak for both models under every formulation* ($|r| < 0.15$, $p > 0.12$), consistent with nWBV being a coarse SIENAX-derived global tissue fraction whose per-subject change is noise-dominated over ∼1–2 year intervals. The discriminating endpoint is agreement magnitude: HypEReg-TransMorph has ∼2× lower MAE/RMSE in every formulation. Sign agreement with ΔnWBV is 0.81–0.84 for both models.

10 OASIS-2 Clinical Association Tests

Table S9: Association between the subject-level Jacobian atrophy proxy and clinical variables on OASIS-2 (linear slopes for continuous covariates; Mann–Whitney for the group contrast). q: Benjamini–Hochberg FDR across the five tests per model.

Model	Test	Statistic	p	q (BH-FDR)
HypEReg-TransMorph	slope vs. Age	2.86×10^{-4}	0.636	0.636
	slope vs. MMSE	1.01×10^{-3}	0.392	0.636
	slope vs. CDR	-2.18×10^{-2}	0.040	0.161
	Demented vs. Nondemented	U=1030	0.617	0.636
TransMorph	slope vs. Age	1.59×10^{-4}	0.886	0.886
	slope vs. MMSE	1.36×10^{-3}	0.534	0.886
	slope vs. CDR	-7.24×10^{-3}	0.712	0.886
	Demented vs. Nondemented	U=853	0.525	0.886

Atrophy proxy is the subject-mean brain-mask $\log\det J_\phi$. HypEReg-TransMorph shows a nominal CDR-graded trend ($p = 0.040$) that does not survive FDR; no other association is significant for either model. These are exploratory associations, not validated biomarkers.

11 Inverse-Consistency Error (IXI)

Table S10: Inverse-consistency error (ICE, in voxels) of the composed forward–backward warp over the 115 IXI test pairs. Lower is better; this is a topology check beyond counting non-positive Jacobian voxels.

Model	ICE mean	ICE std	ICE median	ICE p95
HypEReg-TransMorph	**1.664**	0.101	1.669	1.815
TransMorph	4.207	0.117	4.213	4.380
MIDIR	2.013	0.119	2.000	2.214

ICE is the mean residual displacement magnitude of $\phi_{A\to B} \circ \phi_{B\to A}$ relative to identity, in voxel units, averaged over the brain region. HypEReg-TransMorph attains the lowest ICE despite using no explicit inverse-consistency loss, and is more cycle-consistent than the structurally fold-free MIDIR.

12 Jacobian Discretization Sensitivity

Table S11: Sensitivity of the non-positive Jacobian ratio and SDlogJ to the Jacobian discretization scheme, computed on the same cached IXI displacement fields ($n = 115$ per model). Schemes vary the finite-difference stencil (forward/central), the boundary mask (interior/edge-padded), and the evaluation resolution (full/half).

Model	Scheme (diff / mask / res)	$\det(J_\phi) \leq 0$ ratio	SDlogJ
HypEReg-TransMorph	forward / interior / full	3.39×10^{-5}	0.346
	central / interior / full	1.51×10^{-5}	0.333
	forward / padded / full	3.28×10^{-5}	0.341
	forward / interior / half	4.11×10^{-2}	0.664
TransMorph	forward / interior / full	1.88×10^{-2}	0.520
	central / interior / full	1.55×10^{-2}	0.508
	forward / padded / full	1.82×10^{-2}	0.513
	forward / interior / half	8.31×10^{-2}	0.688
MIDIR	forward / interior / full	3.85×10^{-9}	0.313
	central / interior / full	0	0.301
	forward / padded / full	3.73×10^{-9}	0.308
	forward / interior / half	2.44×10^{-2}	0.693

Model ranking (HypEReg-TransMorph < TransMorph; MIDIR lowest) is preserved under every scheme. Boundary handling is negligible; central differencing slightly lowers all counts (so main-text forward-difference values are mildly conservative); half-resolution inflates all folding statistics by one to three orders of magnitude, motivating full-resolution evaluation with a single shared implementation across models. The full Jacobian (all nine partial derivatives) is used throughout.

13 Multi-Seed Training Variability (IXI)

Table S12: Per-seed and across-seed training variability of TransMorph and HypEReg-TransMorph on the IXI test set. Both models were retrained from scratch under three global seeds $\{0, 1, 2\}$ with identical 403/58/115 split, optimizer (Adam + AMSGrad), 150-epoch schedule, loss weights, and best-validation-Dice checkpoint selection; all six checkpoints were scored with one shared deterministic test pipeline ($n = 115$). Per-seed rows report test mean $\pm$ cross-subject std over 115 subjects; summary rows report mean $\pm$ std across the three per-seed test means.

**Model**	**Seed**	**Dice (17-group)↑**	**$\det(J_\phi) \leq 0$ ratio↓**	**SDlogJ↓**
TransMorph	0	0.7497 ± 0.0324	$1.507 \times 10^{-2} \pm 3.38 \times 10^{-3}$	0.5044 ± 0.0355
TransMorph	1	0.7471 ± 0.0320	$1.496 \times 10^{-2} \pm 3.19 \times 10^{-3}$	0.5052 ± 0.0360
TransMorph	2	0.7508 ± 0.0313	$1.508 \times 10^{-2} \pm 3.48 \times 10^{-3}$	0.5041 ± 0.0361
TransMorph	Summary	0.7492 ± 0.0019	$1.504 \times 10^{-2} \pm 6.6 \times 10^{-5}$	0.5046 ± 0.0005
HypEReg-TransMorph	0	0.7515 ± 0.0271	$2.05 \times 10^{-5} \pm 7.51 \times 10^{-6}$	0.3340 ± 0.0218
HypEReg-TransMorph	1	0.7526 ± 0.0275	$1.62 \times 10^{-5} \pm 6.77 \times 10^{-6}$	0.3271 ± 0.0214
HypEReg-TransMorph	2	0.7555 ± 0.0271	$1.74 \times 10^{-5} \pm 7.15 \times 10^{-6}$	0.3294 ± 0.0215
HypEReg-TransMorph	Summary	**0.7532 ± 0.0020**	$1.80 \times 10^{-5} \pm 2.2 \times 10^{-6}$	**0.3302 ± 0.0035**

In per-seed paired Wilcoxon tests on the same 115 subjects, the HypEReg Dice advantage is significant for seeds 1 and 2 (median $\Delta \approx +0.0035$–0.0038, $p < 10^{-9}$) but not for seed 0 (median $\Delta \approx -0.0004$, $p = 0.24$); the regularity gains are overwhelmingly significant in every seed (non-positive Jacobian ratio and SDlogJ, $p \approx 1.3 \times 10^{-20}$). These TransMorph runs are same-budget, same-hardware retrainings; their test means differ from the released-checkpoint values in main-text Table 2 by less than the across-seed spread.

14 Large-Sample Zero-Shot ROI Jacobian Plausibility ($n = 393$ OASIS pairs)

The official Learn2Reg OASIS test split contains only n=19 pairs, which limits the precision of cross-cohort *overlap* estimates (main-text Section 4.6). To verify that the zero-shot *regularity* advantage of HypEReg-TransMorph is not an artifact of this small sample, we additionally evaluated per-ROI Jacobian plausibility on **393 consecutive labeled cross-subject pairs** drawn from the fully labeled Learn2Reg OASIS set (394 subjects, $p_i \to p_{i+1}$), under the *same* strict IXI→OASIS zero-shot transfer (no OASIS fine-tuning). For each pair we compute the full-resolution Jacobian determinant and, within three atrophy-sensitive ROIs (hippocampus, lateral ventricles, cortical ribbon, Learn2Reg label scheme), the per-ROI non-positive Jacobian ratio and SDlogJ. The two IXI-trained checkpoints (HypEReg-TransMorph and plain TransMorph) differ only in the HypEReg regularizer, so this is a matched comparison. Across all three ROIs and both regularity metrics (six paired contrasts in total), HypEReg-TransMorph reduces folding and log-Jacobian dispersion by roughly one to two orders of magnitude, with every paired Wilcoxon signed-rank test surviving Benjamini–Hochberg correction at $q < 10^{-60}$ (Table S13). This large-sample result reproduces, at $\sim 20\times$ the official sample size, the per-ROI plausibility advantage that the n=19 zero-shot experiment can only suggest.

Table S13: Large-sample IXI→OASIS zero-shot per-ROI Jacobian plausibility on n=393 consecutive labeled cross-subject pairs (394 subjects). Both models are IXI-trained checkpoints evaluated without OASIS fine-tuning; they differ only in the HypEReg regularizer. Lower is better for both metrics.

**ROI**	**$\det(J_\phi) \leq 0$ (HypEReg)↓**	**$\det(J_\phi) \leq 0$ (TransMorph)↓**	**SDlogJ (HypEReg)↓**	**SDlogJ (TransMorph)↓**
Hippocampus	$7.0 \times 10^{-5} \pm 6.3 \times 10^{-4}$	$1.70 \times 10^{-2} \pm 1.66 \times 10^{-2}$	0.446 ± 0.081	1.514 ± 0.543
Lateral ventricles	$1.23 \times 10^{-3} \pm 4.90 \times 10^{-3}$	$2.80 \times 10^{-2} \pm 4.66 \times 10^{-2}$	0.562 ± 0.270	1.542 ± 0.919
Cortical ribbon	$3.3 \times 10^{-4} \pm 1.6 \times 10^{-4}$	$4.32 \times 10^{-2} \pm 9.38 \times 10^{-3}$	0.607 ± 0.030	2.437 ± 0.219

HypEReg = HypEReg-TransMorph; TransMorph = plain TransMorph; both IXI-trained, evaluated zero-shot. Values are mean ± std over n=393 consecutive labeled OASIS cross-subject pairs. All six paired Wilcoxon signed-rank contrasts (HypEReg vs. TransMorph, per ROI and metric) favor HypEReg and survive Benjamini–Hochberg FDR correction at $q < 10^{-60}$. This analysis isolates deformation regularity (folding/SDlogJ); it is complementary to, not a replacement for, the official n=19 Learn2Reg overlap benchmark reported in main-text Table 4.

15 ADNI-Style ROI Clinical-Pattern Validation (OASIS-2)

Following the ADNI tensor-based morphometry convention of prioritizing regional Jacobian patterns and clinical gradients over a single whole-brain correlation, we summarized ROI-integrated Jacobian readouts against CDR, MMSE, and CDR-stratified subject groups. For each model seed, ROI change was averaged to subject level, correlated with CDR/MMSE, and compared between subjects with CDR>0 and CDR$=0$. Table S14 reports across-seed means and significant-seed counts.

Table S14: ADNI-style ROI clinical-pattern summaries on OASIS-2. CDR and MMSE columns report Pearson r between subject-level ROI-integrated Jacobian relative change and the clinical variable; Δ_{CDR} is the median ROI change in CDR>0 subjects minus CDR$=0$ subjects. Values are across-seed mean ± std over three IXI-trained zero-shot checkpoints per model family.

(A) HypEReg-TransMorph

**ROI**	**CDR r**	**MMSE r**	**Δ_{CDR}**	**Sig. seeds (CDR/MMSE)**
Lateral ventricles	0.179 ± 0.051	-0.174 ± 0.034	0.0025 ± 0.0085	2/3 / 2/3
Hippocampus	0.128 ± 0.041	-0.189 ± 0.015	-0.0002 ± 0.0062	0/3 / 1/3
White matter	0.060 ± 0.042	-0.144 ± 0.025	-0.0056 ± 0.0014	0/3 / 0/3
Thalamus	0.163 ± 0.038	-0.158 ± 0.069	0.0052 ± 0.0078	1/3 / 1/3

(B) TransMorph

**ROI**	**CDR r**	**MMSE r**	**Δ_{CDR}**	**Sig. seeds (CDR/MMSE)**
Lateral ventricles	0.113 ± 0.021	-0.131 ± 0.020	0.0013 ± 0.0012	0/3 / 0/3
Hippocampus	0.123 ± 0.046	-0.146 ± 0.025	0.0211 ± 0.0216	0/3 / 0/3
White matter	0.109 ± 0.012	-0.169 ± 0.025	-0.0018 ± 0.0040	0/3 / 0/3
Thalamus	0.171 ± 0.069	-0.213 ± 0.063	0.0170 ± 0.0167	1/3 / 2/3

"Sig. seeds" counts model-specific seeds with BH-FDR-corrected $q < 0.05$ for the corresponding ROI/clinical-variable association. These tests are exploratory because OASIS-2 clinical effects are weak and the models were not trained for clinical endpoint prediction. They are included to align the validation with regional morphometry practice, where local Jacobian patterns and clinical gradients are more informative than a single whole-brain nWBV correlation.

16 Compact Native nWBV Sanity Summary (OASIS-2)

Table S15: Compact subject-level native ΔnWBV/year sanity check moved from the main text. Values are across-seed mean ± std over three IXI-trained zero-shot checkpoints per model family. This table is retained as a coarse global check only; the full robustness panel is reported in Table S8.

**Model**	**Pearson r↑**	**Cal. MAE↓**	**Cal. RMSE↓**
HypEReg-TransMorph	**0.134 ± 0.037**	**0.00403 ± 0.00001**	**0.00583 ± 0.00003**
TransMorph	0.040 ± 0.077	0.00409 ± 0.00003	0.00587 ± 0.00001

Pearson r is computed at subject level (n=150) between the brain-mask Jacobian atrophy proxy and native ΔnWBV/year. The weak correlations support treating nWBV as a coarse sanity endpoint rather than primary morphometric validation.

17 Additional FastSurfer Small-Scale Sanity Validation (OASIS-2)

Table S16: Independent sanity checks for FastSurfer-derived OASIS-2 longitudinal ROI rates. A fixed-seed random subset (n = 60 pairs) is used for small-scale auditing, with full-cohort values (n = 223 pairs) shown for consistency.

(A) Direction consistency on atrophy pairs

**ROI**	**Subset** (n=60)	**Full** (n=223)
Lateral ventricles	46/48 (95.8%); p=4.2×10^{-12}	177/181 (97.8%); p=1.4×10^{-47}
Cerebral cortex	38/48 (79.2%); p=3.1×10^{-5}	148/181 (81.8%); p=6.9×10^{-19}
White matter	44/48 (91.7%); p=7.6×10^{-10}	156/181 (86.2%); p=1.2×10^{-24}
Hippocampus	46/48 (95.8%); p=4.2×10^{-12}	165/181 (91.2%); p=1.2×10^{-32}

(B) Bilateral rate consistency

**ROI**	**Subset** (n=60)	**Full** (n=223)
Lateral ventricles	r=0.888; p=3.4×10^{-21}	r=0.913; p=4.7×10^{-88}
Hippocampus	r=0.838; p=6.7×10^{-17}	r=0.732; p=1.0×10^{-38}

(C) External consistency vs. ΔnWBV/year

**ROI**	**Subset** (n=60)	**Full** (n=223)
Lateral ventricles	r=−0.638; p=4.3×10^{-8}	r=−0.603; p=1.7×10^{-23}
Cerebral cortex	r=0.461; p=2.1×10^{-4}	r=0.396; p=8.3×10^{-10}

Subset sampling uses fixed random seed 2026. Panel (A): one-sided binomial tests against 0.5. Panels (B–C): Pearson correlations. These checks are segmentation-only plausibility audits, independent of registration-model identity.
