# Supplementary figures and images for: Hyperelastic Regularization for Near-Diffeomorphic Transformer-Based Brain MRI Registration
Source: J Imaging. 2026 Jun 24;12(7):276. doi: 10.3390/jimaging12070276 (PMC13413009; doi:10.3390/jimaging12070276)

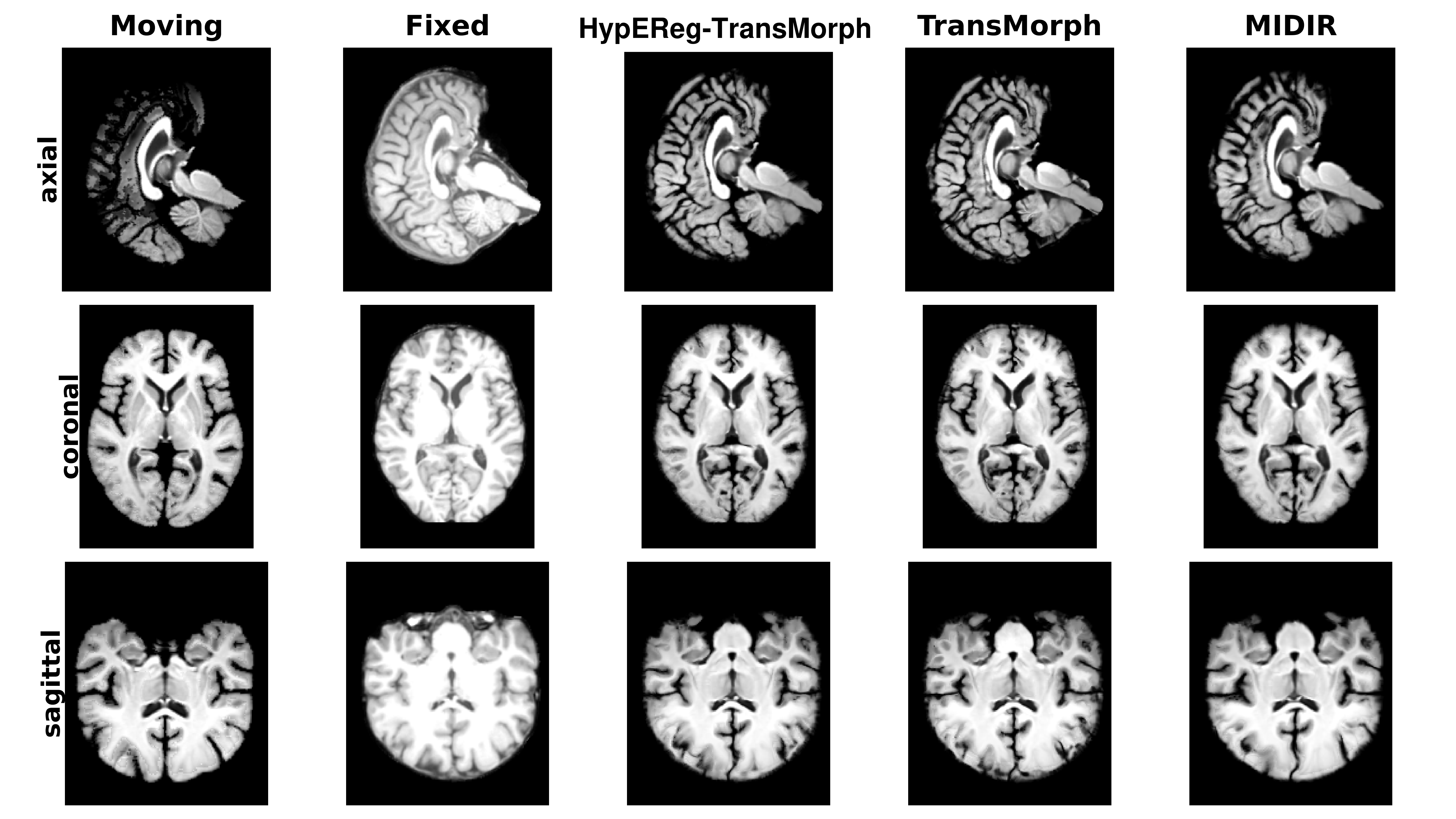

Supplement: Supplementary file 1 [file jimaging-12-00276-s001.zip › figures/fig2_qualitative.png]

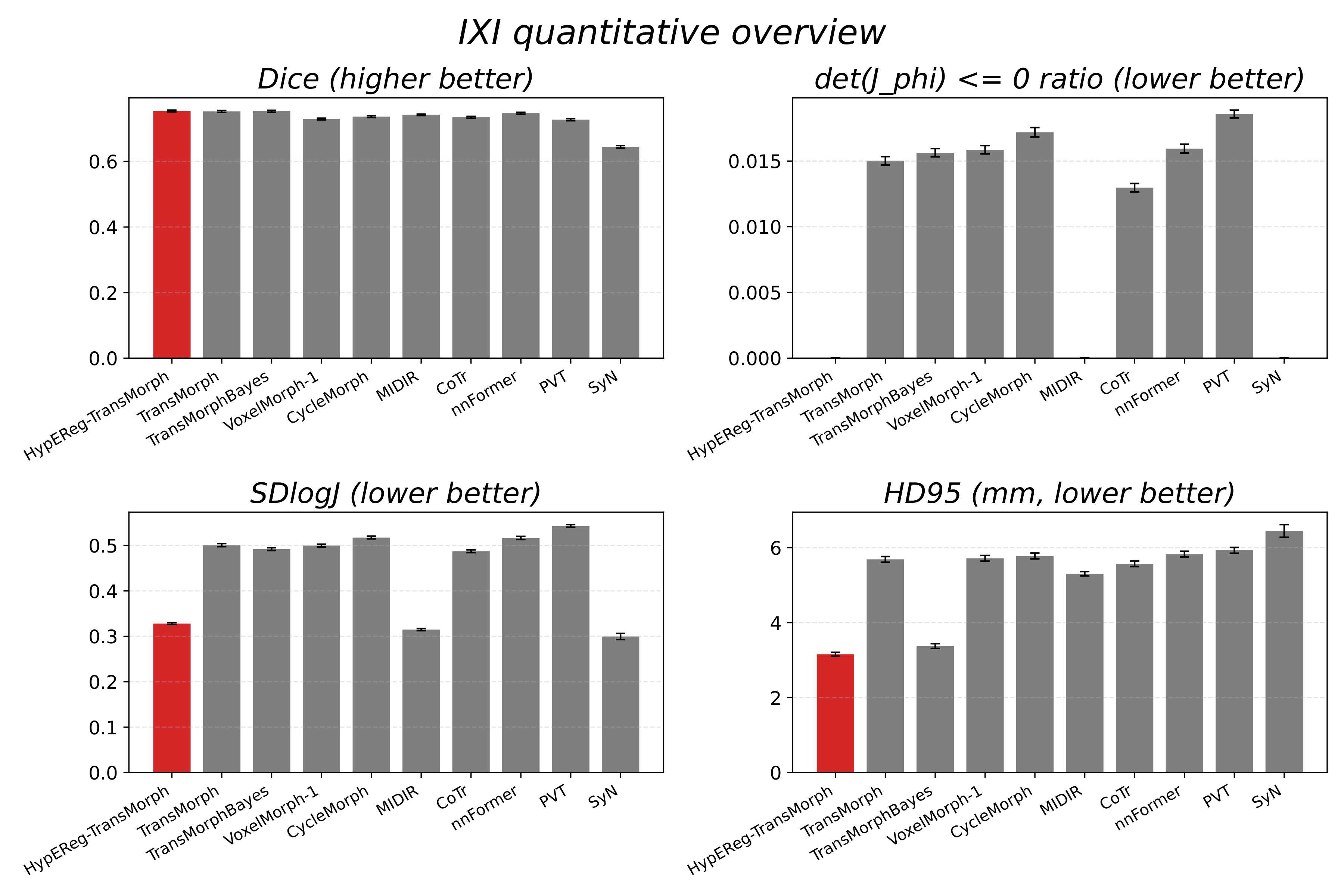

Supplement: Supplementary file 1 [file jimaging-12-00276-s001.zip › figures/fig5_metrics.png]
